# Supplementary material for: Seasonality and mobility: An Integrative framework for reconstructing Kura-Araxes pastoral systems at Maxta I, Nakhchivan
Source: PLoS One. 2026 Apr 16;21(4):e0346108. doi: 10.1371/journal.pone.0346108 (PMC13086362; doi:10.1371/journal.pone.0346108)
Supplement: S1 File — (DOCX) [file pone.0346108.s001.docx]

**S1 File. Environmental Background**

**Spatial variation in the climate of the Caucasus**

***Topography***

Maxta I is situated at 800 meters above sea level in the Sharur Plain, which is bordered by the Lesser Caucasus to the east). The highest peak in the region, Mount Qapichig, stands at 3904 meters. To the west, the Ordubad Plateau, with an average elevation of 1000–1500 meters**^1^**. In the southwest, the Alinja Plain lies at 400 meters, while the Julfa Plain, along the Aras River, is in the northeast. The Caucasus features a diverse range of elevations and is divided into two major mountain ranges: the Greater Caucasus to the north and the Lesser Caucasus to the south. The Lesser Caucasus ranges from 1500 to 2500 meters, with Mount Aragats in Armenia as its highest peak at 4090 meters**^2^**. The Armenian Plateau, spanning Armenia, eastern Turkey, and northwestern Iran, lies at 1,800–2,000 m and contains Lake Van and Lake Urmia**^3^**. To the west, Eastern Anatolia features highlands ranging from 1,000 to 3,000 m. The Greater Caucasus extends from the Black Sea to the Caspian Sea, with an average elevation of 3000 meters. Glaciers are common above 2500 meters**^2^**.

***Temperature***

In the Sharur Plain, present day temperatures vary seasonally (Figure 3). Winters are cold and arid (0°C to -7°C), while summers are hot and dry (30–35°C). Spring and autumn are mild and wetter. The Greater and Lesser Caucasus experience extreme temperature fluctuations, with winters dropping below freezing and prolonged snowfall at higher altitudes. Summer temperatures range from the mid-20s°C to mid-30s°C. The Black Sea’s coastal regions benefit from its moderating influence, resulting in milder winters and summers compared to inland areas**^4^** .

***Rainfall***

Present day rainfall is the heaviest in spring and autumn, while summers are the driest, but exhibit the highest variability in northern Eurasia**^5^** (Figure 3). Thunderstorms frequently occur at higher elevations during summer afternoons. The coastal plains of the Black and Caspian Seas receive the highest annual precipitation. The western slopes of the Greater Caucasus receive more rainfall than the eastern slopes. In contrast, the southern Caucasus (Azerbaijan and Armenia) remains drier due to the Lesser Caucasus blocking moisture-laden winds. In general, mountainous regions receive more precipitation than lowlands**^6^**.

***Snowfall***

From November to April, the mountains surrounding Maxta I, including the Lesser Caucasus and Eastern Anatolian Highlands, receive substantial snowfall (Figure 3). The Greater Caucasus captures moisture from prevailing westerly winds, leading to heavy snow accumulation**^7^**. At elevations above 2,000 m, snow depths reach several meters, contributing to glacier formation and sustaining regional river headwaters. In alpine and sub-alpine zones, the snowpack functions as a natural reservoir, gradually releasing meltwater**^8,9^**. Meltwater from the Lesser Caucasus feeds rivers around Maxta in Nakhchivan. The Sharur plain experiences milder winters, with occasional but less severe snowfall.

**Spatial and seasonal variation in oxygen isotopes in the Caucasus**

***δ^18^O values in precipitation, lakes, rivers and springs***

The δ^18^O (V-SMOW) values of present day precipitation at Maxta I vary from -15.4‰ in the winter to -1.8‰ in the summer**^10^** (Supplementary material 1). The Kura and Aras rivers are the primary waterways in the Caucasus. The Aras River and its tributaries, such as the Arpaçay, feed the Sharur Plain (Maxta I) (Supplementary material 1). Glaciers in the Caucasus provide seasonal freshwater, sustaining river flow during warmer months**^8^**. The region contains several major freshwater lakes, including Lake Sevan in Armenia and Lake Çıldır in eastern Turkey. Groundwater is stored in various aquifers, accessible through wells or natural springs**^11^**. Reported δ^18^O values in regional freshwater sources range from -19.0‰ to -8.1‰ (Supplementary material 1).

***The effects of different atmospheric circulation on δ^18^O values in precipitation***

The North Atlantic Oscillation (NAO) is a pattern of atmospheric circulation that has a major influence on the strength and position of mid-latitude westerlies in the Northern Hemisphere. This is particularly relevant to the δ^18^O values of precipitation. During negative NAO phases, the westerlies are weakened, allowing precipitation enriched with heavy isotopes from the Mediterranean and southerly sources to reach the Armenian Highlands. On the other hand, positive NAO phases are associated with stronger westerlies, which bring more negative δ^18^O values in precipitation from the Black Sea. This relationship is most pronounced during the winter and spring months (December to May)**^12^** .

***δ^18^O values along the altitudinal gradient***

It has been argued**^12^** that elevation has no significant effect on δ^18^O values in the Armenian Highlands.

**Spatial and seasonal variation in floral δ^13^C values in the Caucasus**

***Local & regional vegetation***

The Caucasus is highly varied in its C_3_ and C_4_ plant composition. A detailed description of the vegetation of the Caucasus is given in Supplementary material 2. Maxta I is located in a semi-arid steppe or arid steppe environment (Supplementary material 2). The arid steppes are dominated by C_3_ grasses in the cooler months. This vegetation zone in the warmer months also supports C_4_ chenopods (Supplementary material 2). With increasing altitude, semi-deserts become more steppe-like featuring more pure C_3_ grasses. Semi-arid steppe environments are dominated by C_3_ grasses in cooler months such as *Stipa sp.* and *Festuca sp.* and C_4_ grass such as *Bothriochloa* in warmer months (Supplementary material 2). Higher elevation sub- and alpine meadows are dominated by pure C_3_ communities (Supplementary material 2). C_4_ plants in the Caucasus, Iran, and Eastern Anatolia are primarily found in sandy deserts, halophytic environments, grasslands, and ruderal or disturbed habitats**^13^**. Their distribution is shaped by environmental factors such as temperature, water availability, and soil salinity, with distinct ecological niches across these regions. In sandy desert and coastal dune ecosystems, C_4_ vegetation dominates arid environments. In the central desert of Iran, Haloxylon species form the dominant plant communities, while in the temperate deserts of Iran, sparse grasslands of *Stipagrostis plumosa* are accompanied by *Artemisia* shrubland**^13^**. Along the Caspian shore, coastal dune ecosystems support tall C_4_ grasses**^13^** (Supplementary material 2). Halophytic vegetation in these regions is characterised by C_4_ species that thrive in saline environments, particularly in salt marshes, saline flats, and inland salt plains. Dry marly or clayey hills attract C_4_ chenopods, while saline plains, depressions, and salt marshes, such as the Turkman salt flats in Iran, are dominated by these species**^13,14^**. Along the coastal saline flats of the Caspian Sea, salt-tolerant C4 vegetation forms extensive plant communities. Inland saline clayey soils with a high water table, such as those in the Sharur Plain, support pure and mixed stands of *Aeluropus lagopoides,* *Desmostachya bipinnata*, and *Salvadora oleoides***^13^**. C_4_ grasses dominate many grassland ecosystems in these regions, particularly where high temperatures and seasonal summer precipitation create favorable conditions. South-facing slopes of Caspian forests host C_4_ grassland communities**^14^** . The South Caspian C_4_ grasslands belong to the Cymbopogono-Brachypodietalia ramosi order**^15,16^**. In addition to arid and semi-arid environments, C_4_ plants are also found in mesophytic and hydrophytic communities along riverbanks and seasonal watercourses, where high humidity and fluctuating water levels support their growth. Ruderal and weedy C_4_ species are prevalent in disturbed habitats, including agricultural fields, roadsides, and abandoned lands, where they develop rapidly during the summer**^13^** (Supplementary material 2).

***Reported δ^13^C values of C_3_ and C_4_ plants in the Caucasus***

The δ^13^C values of common C_3_**^17,19^** and C_4_**^13^** plants in the Caucasus and their respective values in enamel of herbivores are shown in Supplementary material 2.

***δ^13^C values along the altitude gradient in the Caucasus***

A study**^19^** of modern plant samples around the Kura-Araxes site of Koehne Shahar in the highlands of Northwestern Iran has shown a positive correlation between δ^13^C values of plants and increasing altitude. The authors suggest a +0.46‰ increase per 100 m, which is higher than the previously reported +0.12‰**^18^**. Removing trees from the equation reduces the enrichment rate to 0.36‰ per 100 m.

**References**

1. Thevenin, M. Animal mobility, human mobility: a geopolitical of sheep in Armenia. *Quat. Int.* 579, 99–114 (2021).
2. Saintot, A. *et al.* The Mesozoic–Cenozoic tectonic evolution of the Greater Caucasus. *Geol. Soc. London, Mem.* 32, 277–289 (2006).
3. Dhont, D. & Chorowicz, J. Review of the neotectonics of the Eastern Turkish–Armenian Plateau by geomorphic analysis of digital elevation model imagery. *Int. J. Earth Sci.* 95, 34–49 (2006).
4. Ashabokov, B. *et al.* Climate of the Caucasus region of the last 60 years: precipitation and temperature trends and anomalies. *In* *International Symposium "Engineering and Earth Sciences: Applied and Fundamental Research"*, 716–721 (Atlantis Press, 2019).
5. Kozachek, A. *et al.* Large-scale drivers of Caucasus climate variability in meteorological records and Mt El’brus ice cores. *Clim. Past* 13, 473–489 (2017).
6. Martin-Benito, D., Ummenhofer, C., Köse, N., Güner, H. T. & Pederson, N. Tree-ring reconstructed May–June precipitation in the Caucasus since 1752 CE. *Clim. Dyn.* 47, 3011–3027 (2016).
7. Shahgedanova, M., Popovnin, V., Aleynikov, A., Petrakov, D. & Stokes, C. Long-term change, interannual and intraseasonal variability in climate and glacier mass balance in the central Greater Caucasus, Russia. *Ann. Glaciol.* 46, 355–361 (2007).
8. Hagg, W., Shahgedanova, M., Mayer, C., Lambrecht, A. & Popovnin, V. A sensitivity study for water availability in the Northern Caucasus based on climate projections. *Glob. Planet. Chang.* 73, 161–171 (2010).
9. Perez Leon, N., Bruzzone, O. & Easdale, M. A framework to tackling the synchrony between social and ecological phases of the annual cyclic movement of transhumant pastoralism. *Sustainability* 12, 3462 (2020).
10. Bowen, G. & Revenaugh, J. Interpolating the isotopic composition of modern meteoric precipitation. *Water Resour. Res.* 39, 1299 (2003).
11. Babaev, M., Gurbanov, E. & Ramazanova, F. Main types of soil degradation in the Kura–Aras Lowland of Azerbaijan. *Eurasian Soil Sci.* 48, 445–456 (2015).
12. Brittingham, A. *et al.* Influence of the North Atlantic Oscillation on δD and δ¹⁸O in meteoric water in the Armenian Highland. *J. Hydrol.* 575, 513–522 (2019).
13. Rudov, A., Mashkour, M., Djamali, M. & Akhani, H. A review of C4 plants in southwest Asia: An ecological, geographical and taxonomical analysis of a region with high diversity of C4 eudicots. *Front. Plant Sci.* 11, 546518 (2020).
14. Akhani, H. Biodiversity of halophytic and sabkha ecosystems in Iran. *In* *Sabkha Ecosystems: Volume II: West and Central Asia*, 71–88 (Springer, 2006).
15. Díez-Garretas, B. & Asensi, A. Syntaxonomic analysis of the Andropogon-rich grasslands (Hyparrhenietalia hirtae) in the western Mediterranean region. *Folia Geobot.* 34, 307–320 (1999).
16. Mucina, L. *et al.* Vegetation of Europe: hierarchical floristic classification system of vascular plant, bryophyte, lichen, and algal communities. *Appl. Veg. Sci.* 19, 3–264 (2016).
17. Shishlina, N., Sevastyanov, V. & Kuznetsova, O. Seasonal practices of prehistoric pastoralists from the south of the Russian plain based on the isotope data of modern and archaeological animal bones and plants. *J. Archaeol. Sci. Rep.* 21, 1247–1258 (2018).
18. Körner, C., Farquhar, G. & Roksandic, Z. A global survey of carbon isotope discrimination in plants from high altitude. *Oecologia* 74, 623–632 (1988).
19. Samei, S., Munro, N., Alizadeh, K. & Hartman, G. Highland pastoralism in the Early Bronze Age Kura-Araxes cultural tradition: Stable oxygen (δ¹⁸O) and carbon (δ¹³C) isotope analyses of herd mobility at Köhne Shahar in northwestern Iran. *J. Archaeol. Sci. Rep.* 47, 103773 (2023).
