# Supplementary material for: Seasonality and mobility: An Integrative framework for reconstructing Kura-Araxes pastoral systems at Maxta I, Nakhchivan
Source: PLoS One. 2026 Apr 16;21(4):e0346108. doi: 10.1371/journal.pone.0346108 (PMC13086362; doi:10.1371/journal.pone.0346108)
Supplement: S2 File — (DOCX) [file pone.0346108.s002.docx]

**S2 File. Additional Images**

All images are courtesy of Safar Ashurov and Fidan Khalafova Aliyeva.

|  | 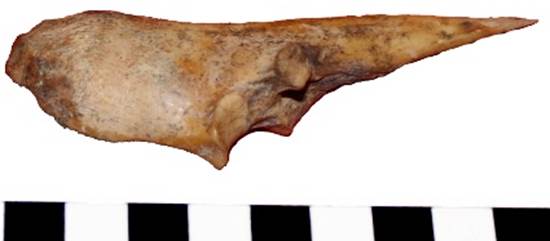 | | 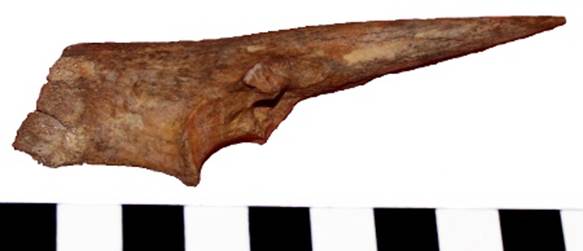 |
| --- | --- | --- | --- |
|  | 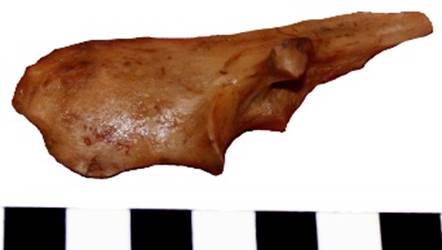 | | 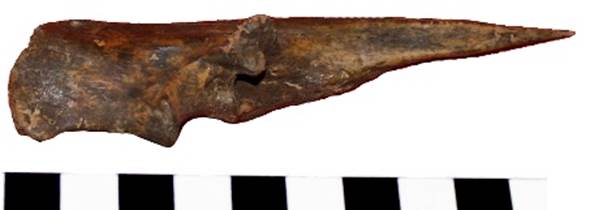 |
|  | 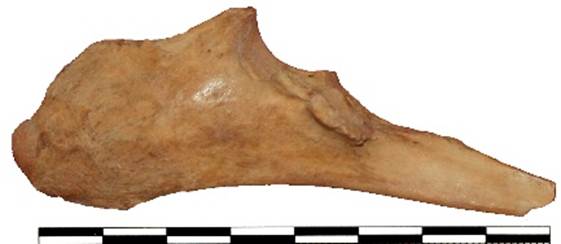 | | 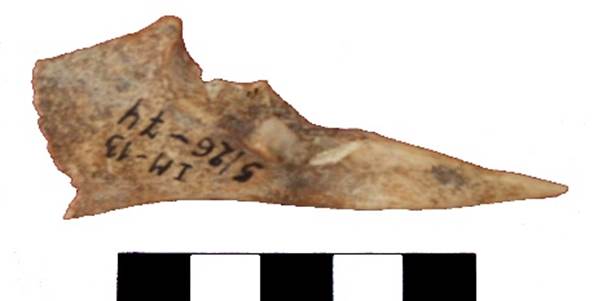 |
| 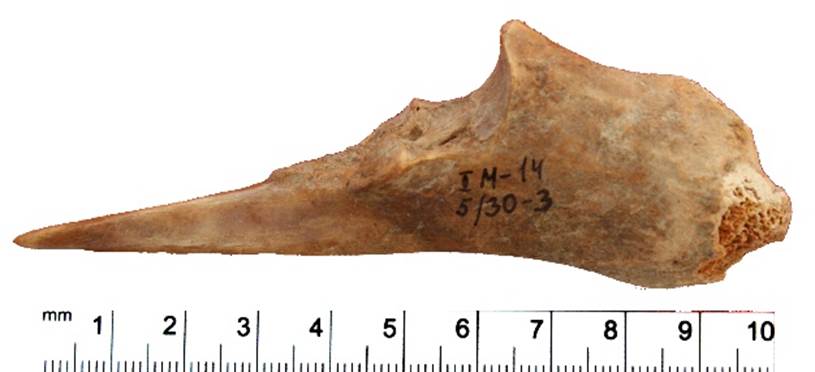 | | 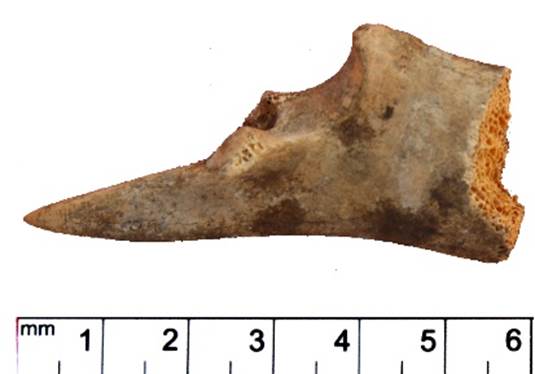 | |
| 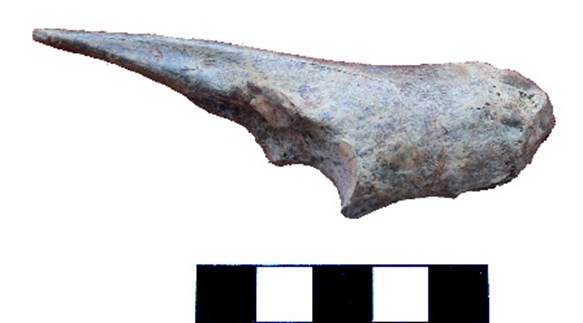 | | 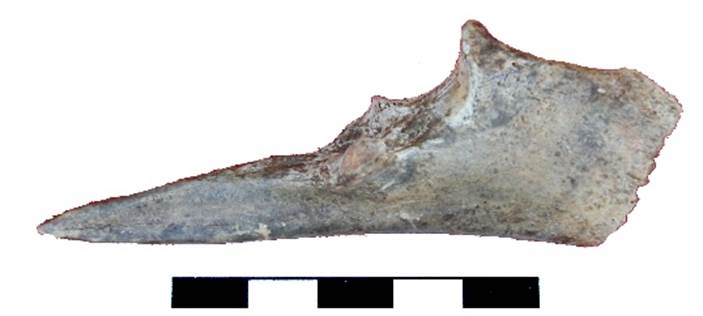 | |
| 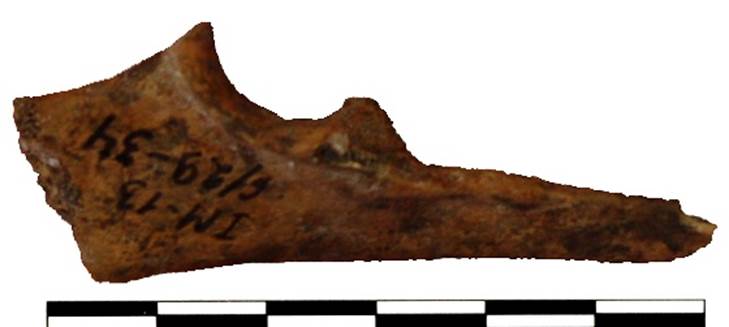 | | 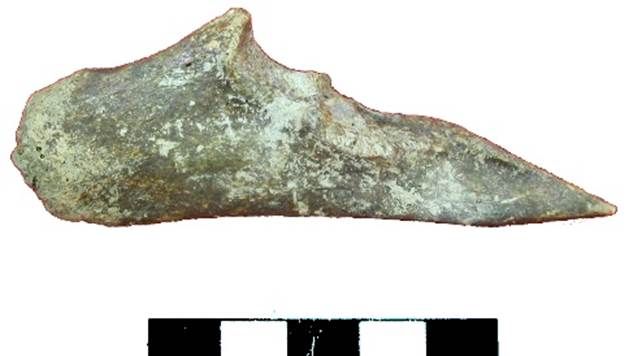 | |
| 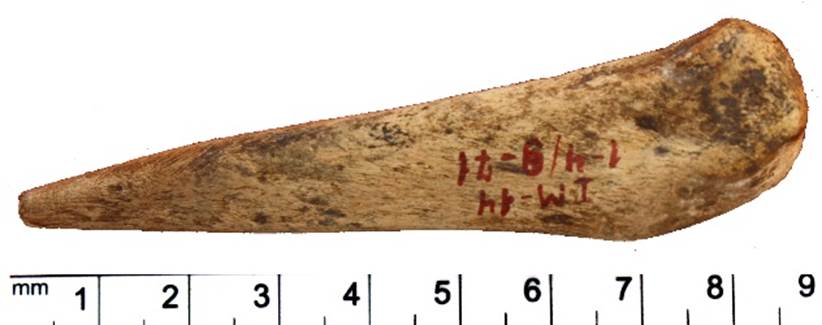 | | 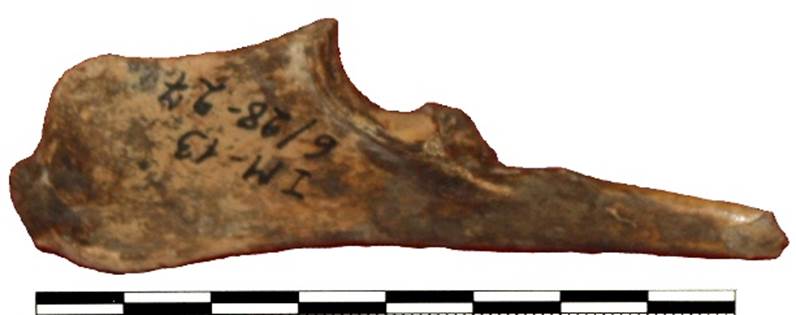 | |
| 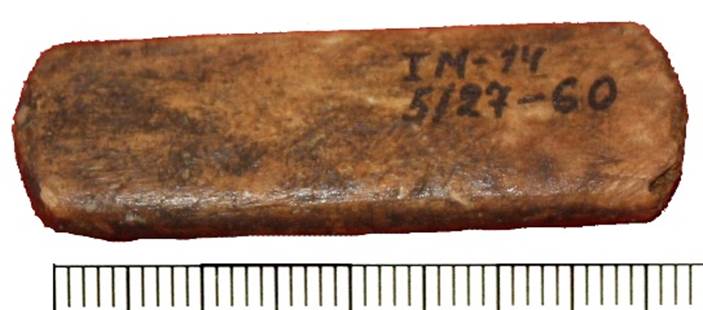 | | 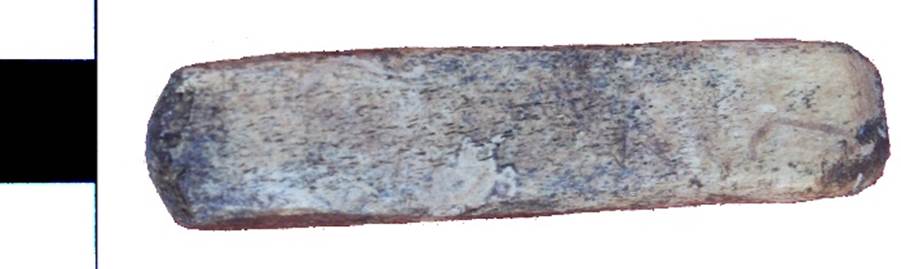 | |
| 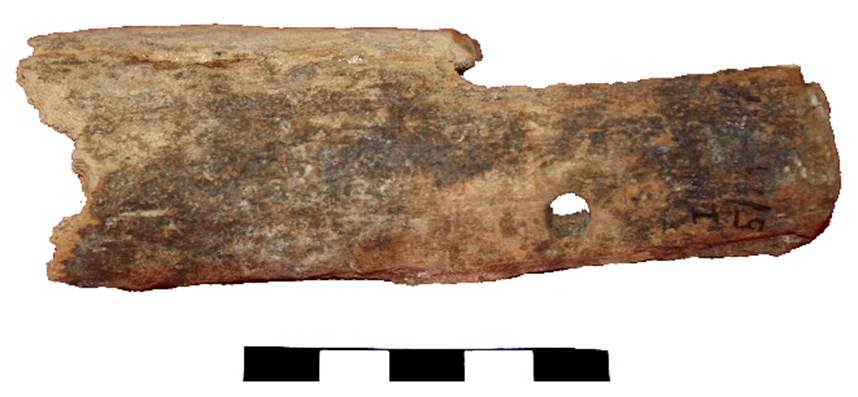 | | 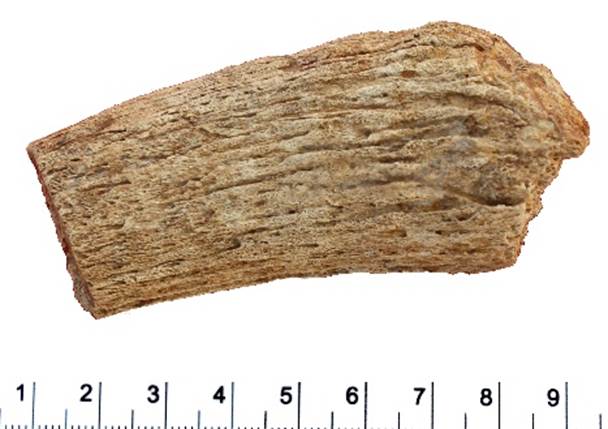 | |
| 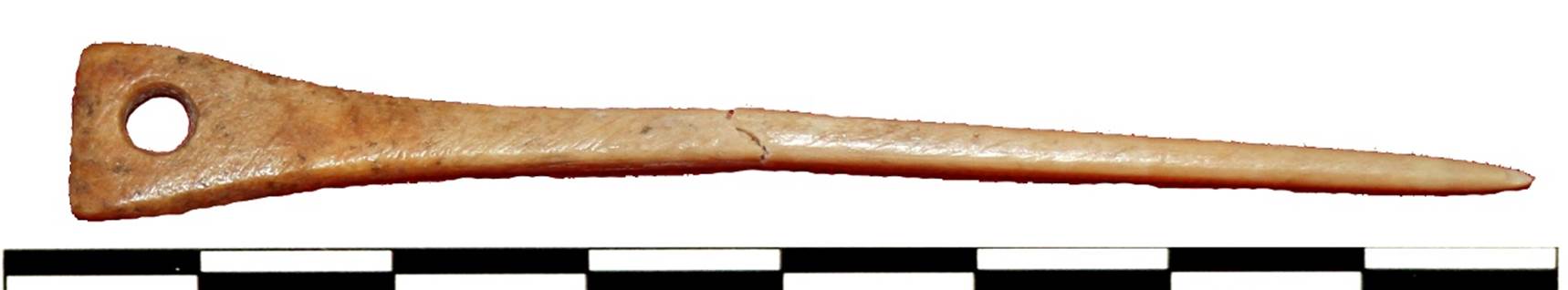 | | | |

**Figure A. Bone tools associated with textile production from Maxta I.** A selection of bone awls, needles, and related implements interpreted as tools used in textile production.

|  | | 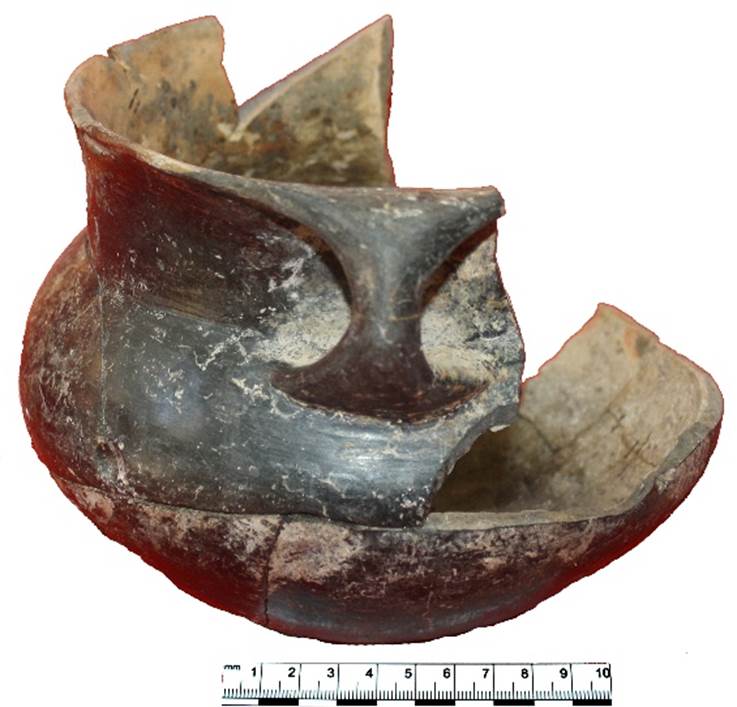 | |
| --- | --- | --- | --- |
| 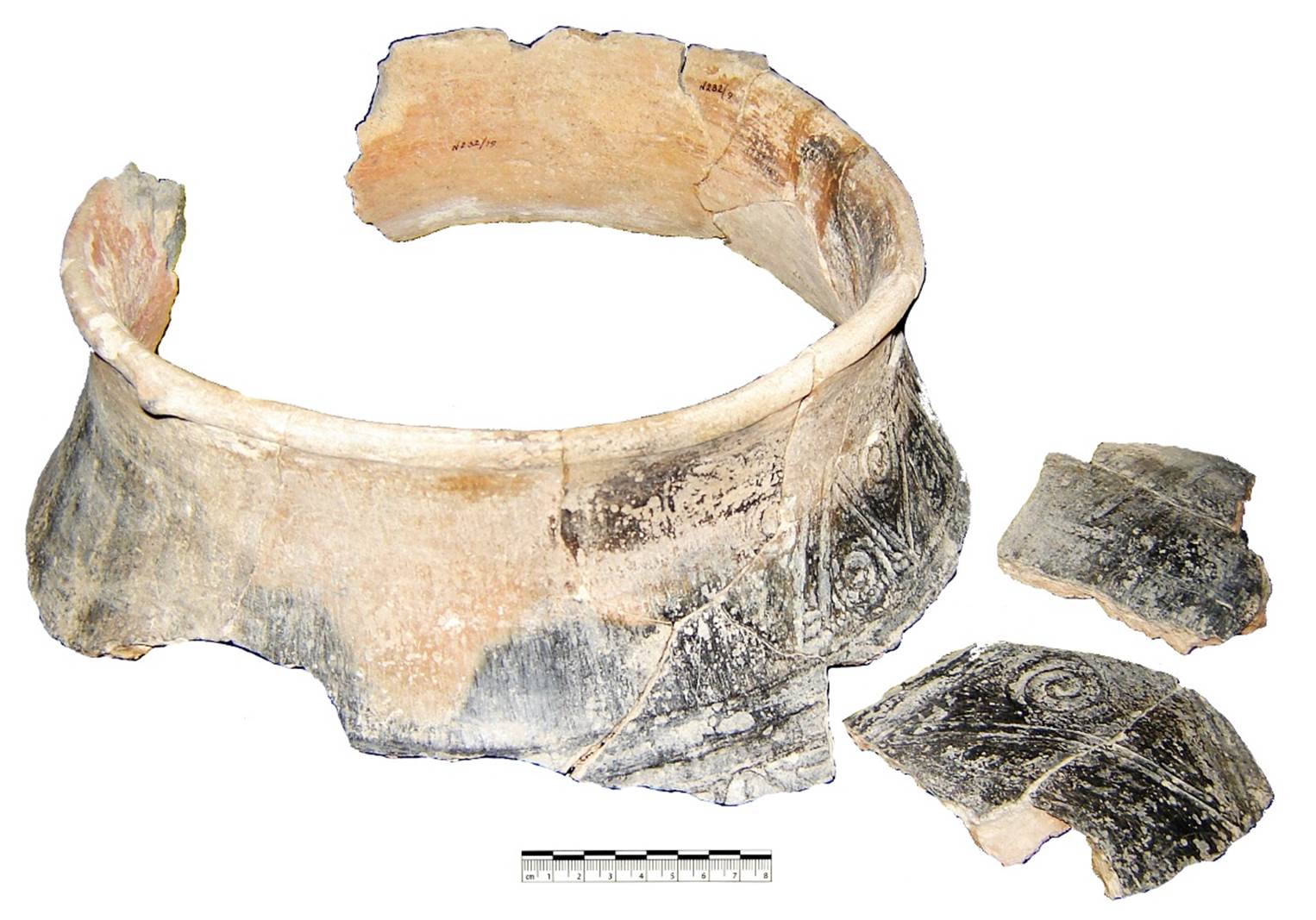 | | | |
| 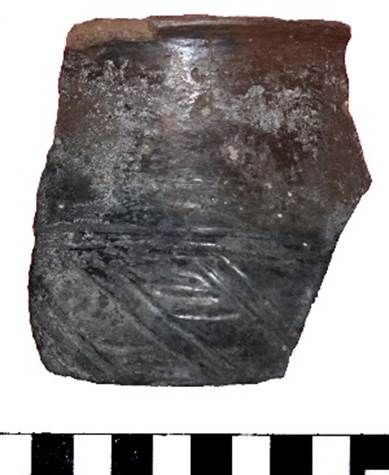 | 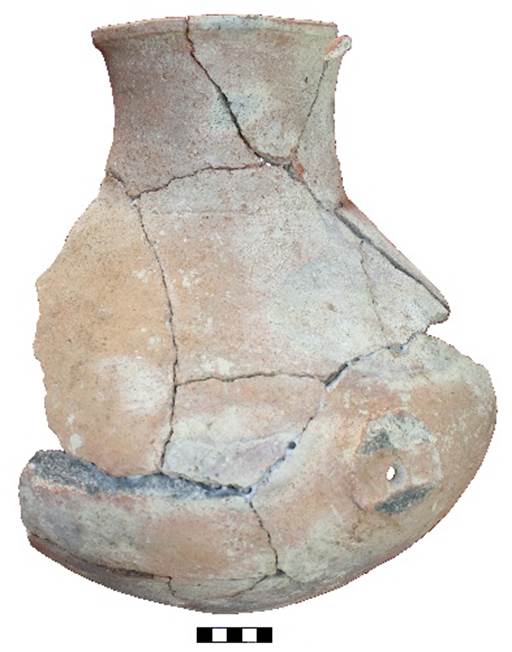 | | 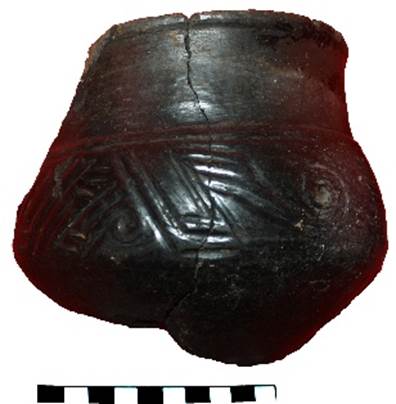 |

**Figure B. Ceramic examples from Maxta I.** Selected ceramic fragments from the Maxta I, illustrating characteristic forms, surface treatments, and decorative styles associated with the region.


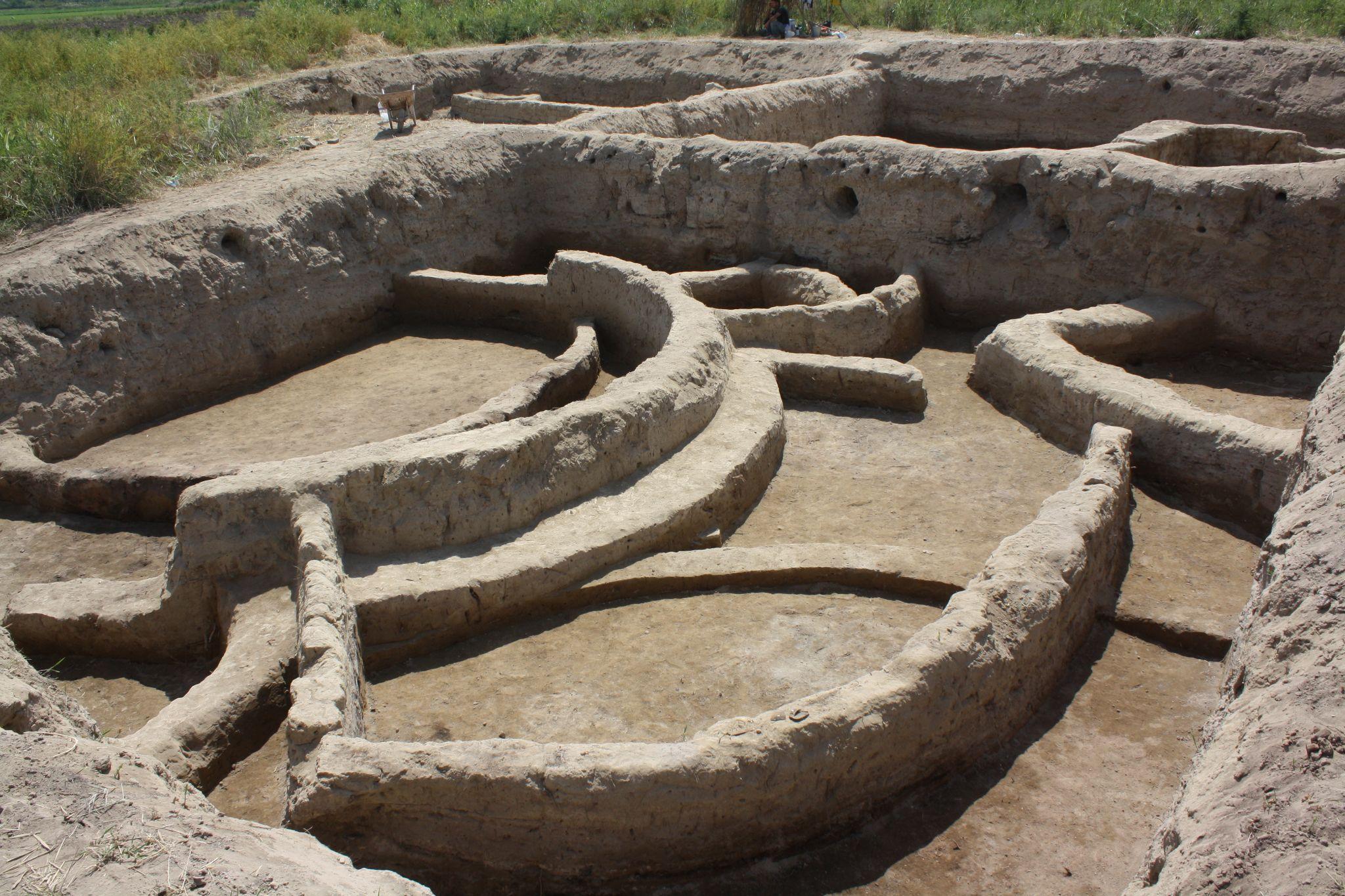


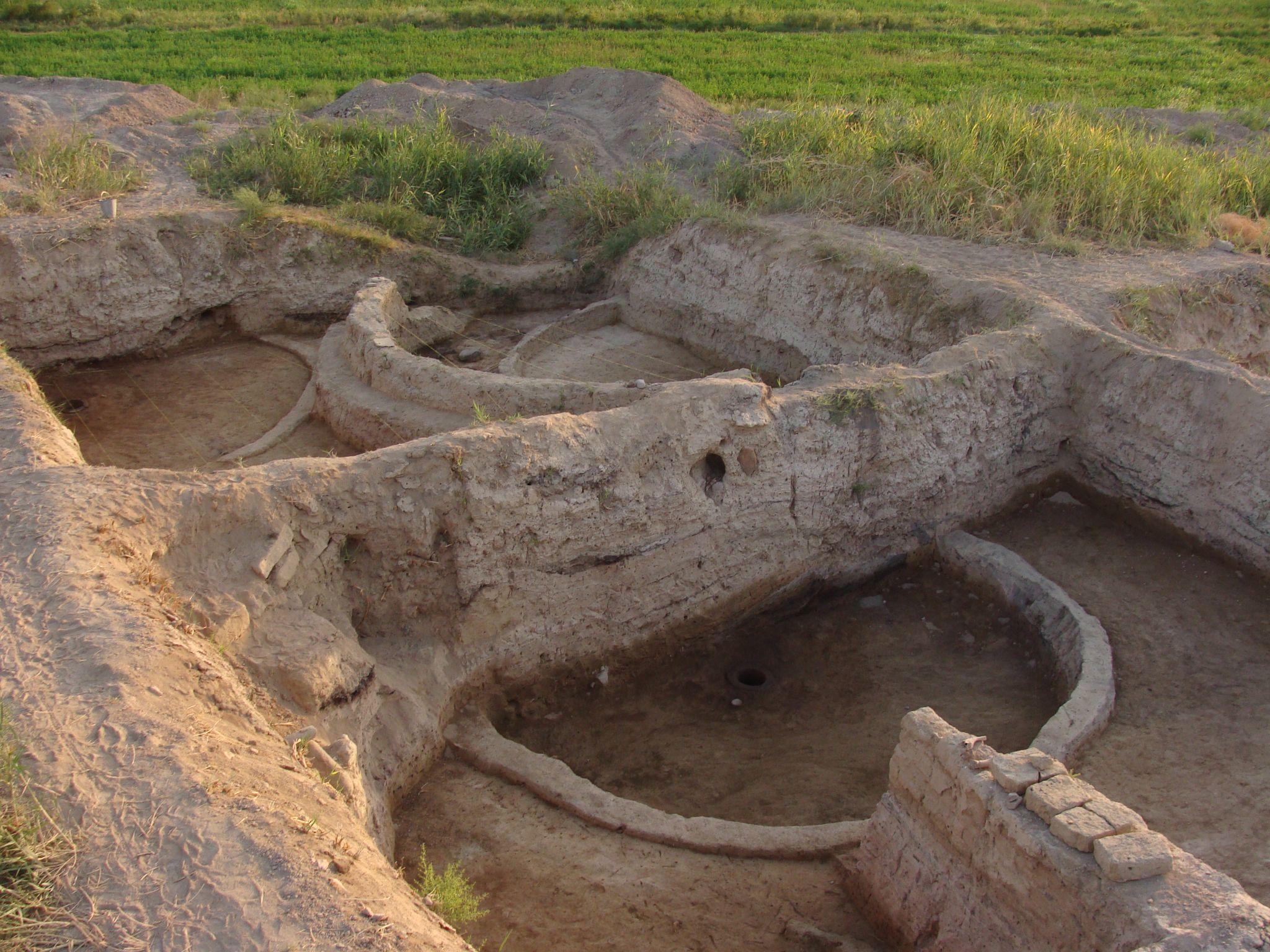


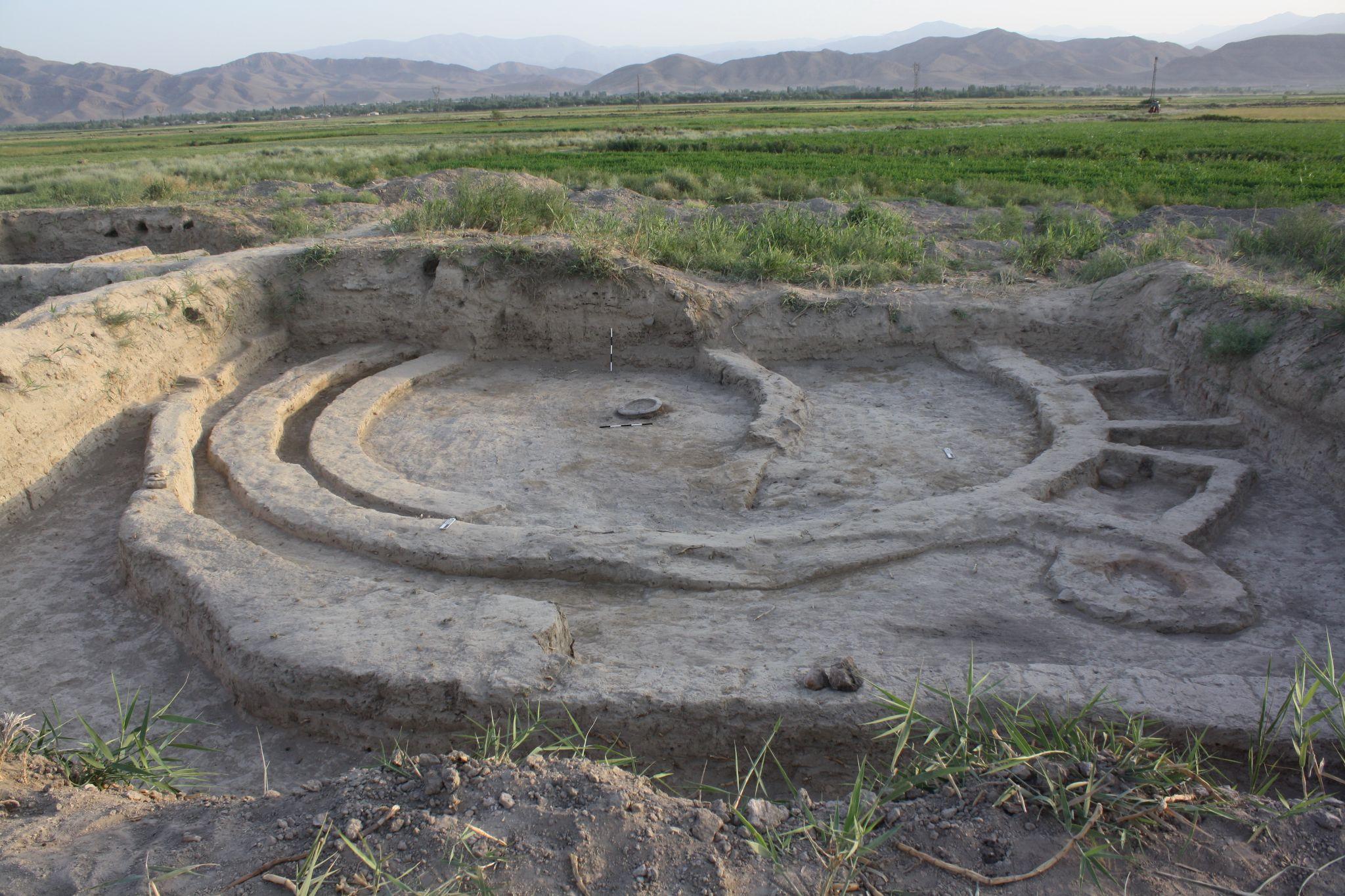


**Figure C. Structures at Maxta I.**

| **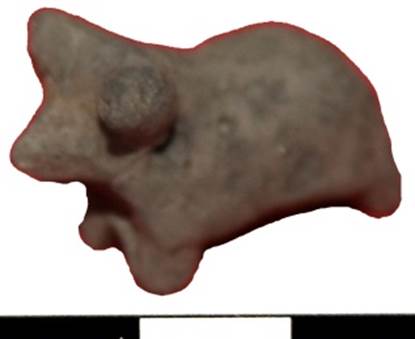** | **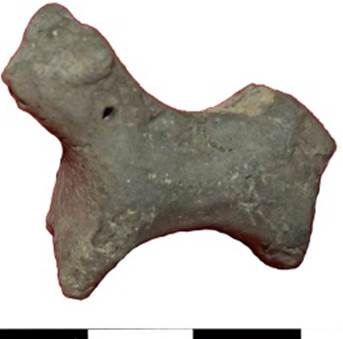** | **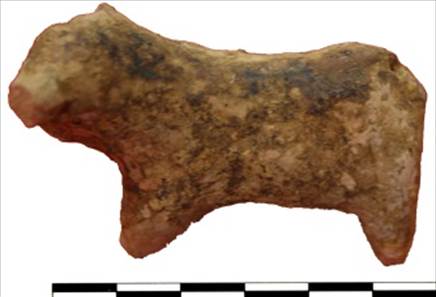** | **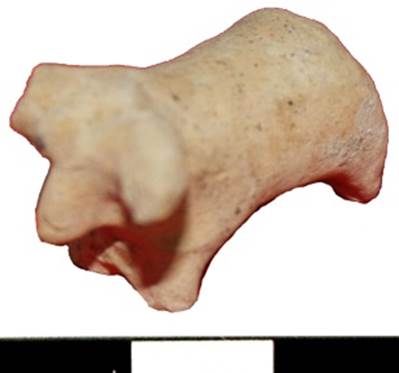** |
| --- | --- | --- | --- |
| **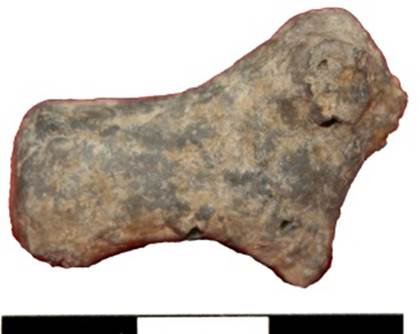** | **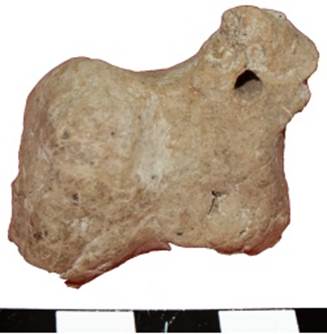** | **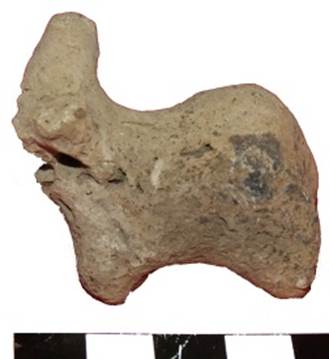** | **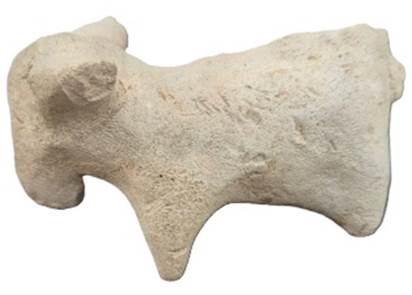** |
| **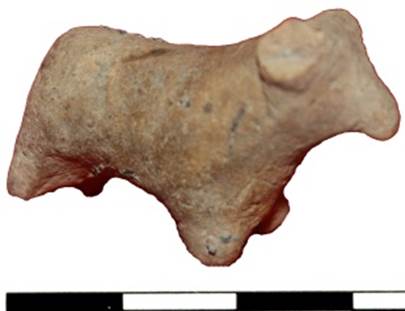** | **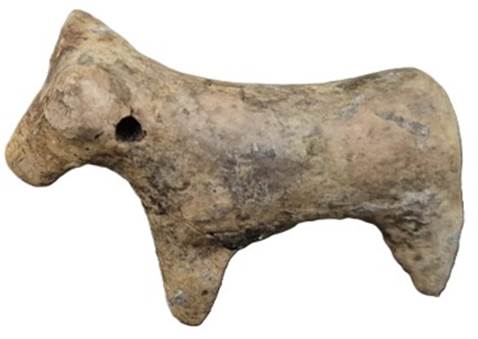** | **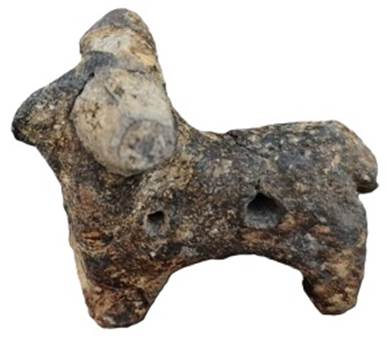** | **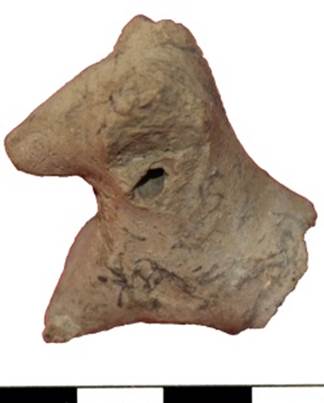** |
| **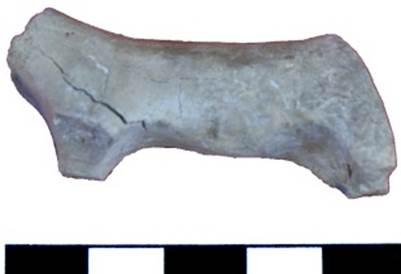** | **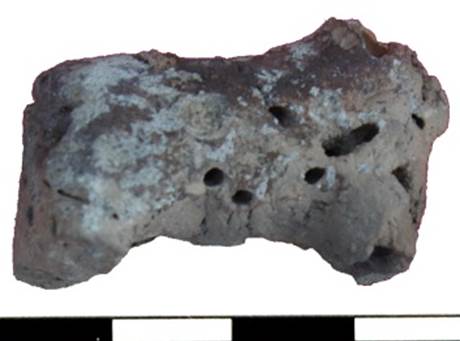** | **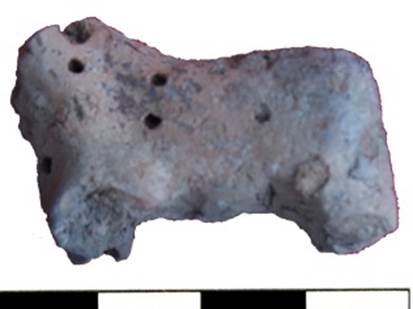** | **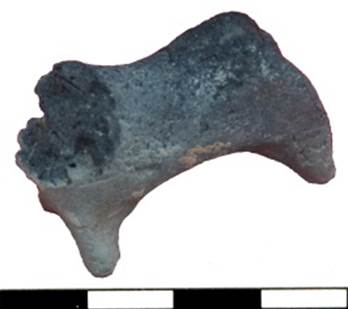** |

**Figure D. Zoomorphic figurines from Maxta I.**
