# Supplementary figures and images for: Seasonality and mobility: An Integrative framework for reconstructing Kura-Araxes pastoral systems at Maxta I, Nakhchivan
Source: PLoS One. 2026 Apr 16;21(4):e0346108. doi: 10.1371/journal.pone.0346108 (PMC13086362; doi:10.1371/journal.pone.0346108)

MI16

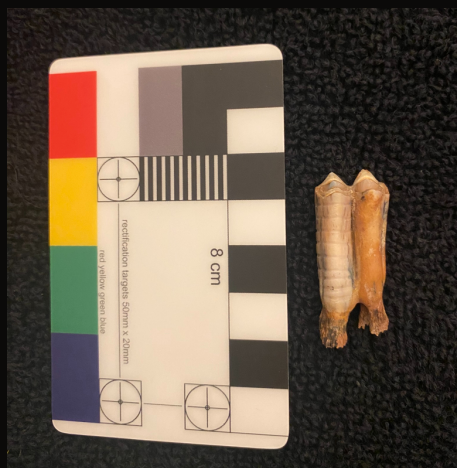

MI18

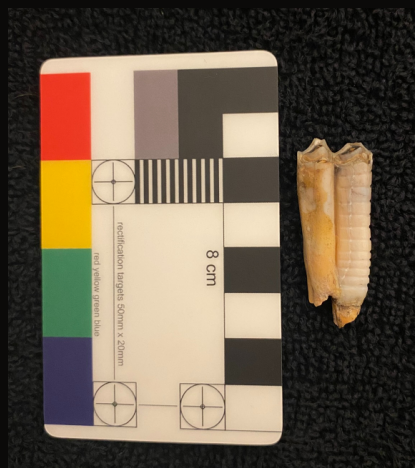

MI21

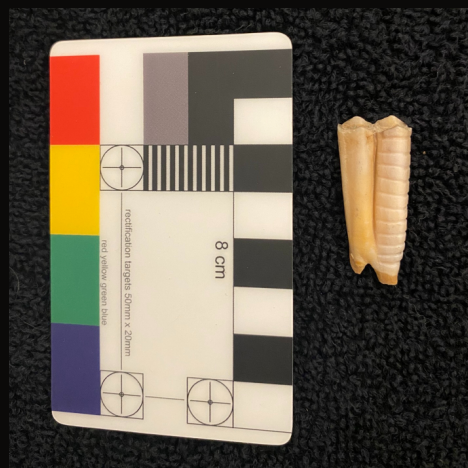

MI25

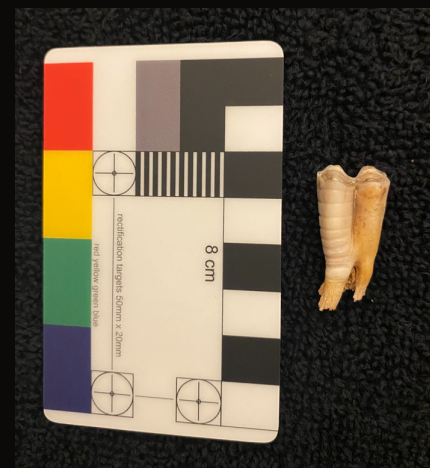

MI42

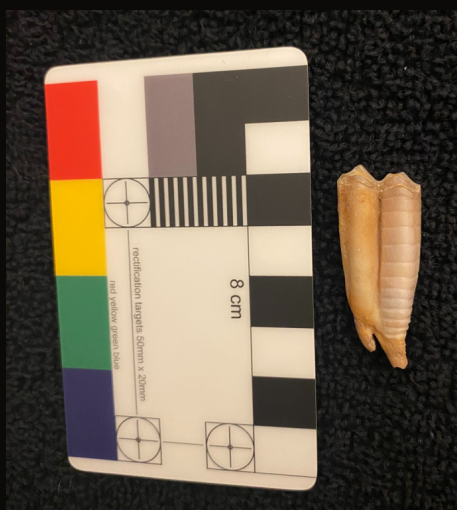

MI46

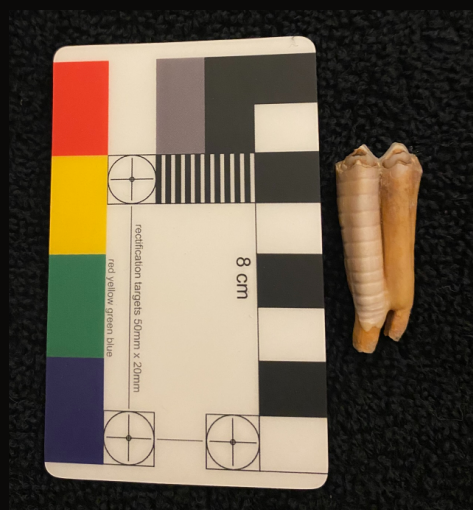

MI47

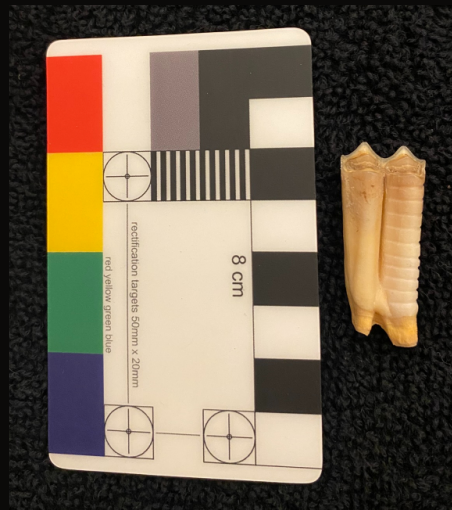

MI53

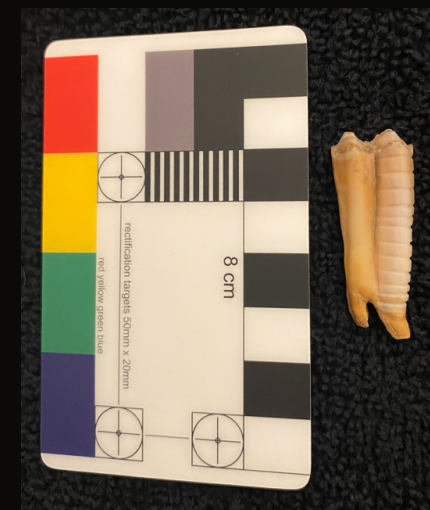

MI57

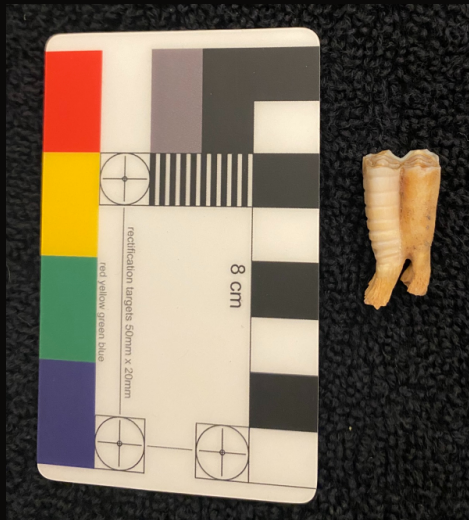

MI59

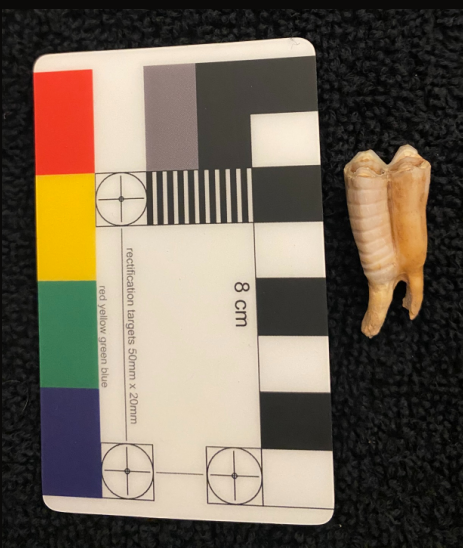

*Note: Lines were measured along the lower ridge*

Supplement: S9 File — (PDF) [file pone.0346108.s009.pdf]
