# Supplementary material for: Seasonality and mobility: An Integrative framework for reconstructing Kura-Araxes pastoral systems at Maxta I, Nakhchivan
Source: PLoS One. 2026 Apr 16;21(4):e0346108. doi: 10.1371/journal.pone.0346108 (PMC13086362; doi:10.1371/journal.pone.0346108)
Supplement: Attachment — Submitted filename: Response to Reviewers.docx [file pone.0346108.s011.docx]

We thank the Academic Editor and the reviewers for their detailed and constructive comments, which have greatly improved our manuscript. Below we provide a detailed response to each point. Reviewer/Editor comments are shown in **black**, followed by our responses in **blue**.

**Journal requirements:**

When submitting your revision, we need you to address these additional requirements.

**1.** Please ensure that your manuscript meets PLOS ONE's style requirements, including those for file naming. The PLOS ONE style templates can be found at

<https://journals.plos.org/plosone/s/file?id=wjVg/PLOSOne_formatting_sample_main_body.pdf> and

<https://journals.plos.org/plosone/s/file?id=ba62/PLOSOne_formatting_sample_title_authors_affiliations.pdf>

We have formatted the manuscript according to the PLOS One guidelines.

**2.** Please include a complete copy of PLOS’ questionnaire on inclusivity in global research in your revised manuscript. Our policy for research in this area aims to improve transparency in the reporting of research performed outside of researchers’ own country or community. The policy applies to researchers who have travelled to a different country to conduct research, research with Indigenous populations or their lands, and research on cultural artefacts. The questionnaire can also be requested at the journal’s discretion for any other submissions, even if these conditions are not met. Please find more information on the policy and a link to download a blank copy of the questionnaire here: [Best Practices in Research Reporting | PLOS One](https://journals.plos.org/plosone/s/best-practices-in-research-reporting). Please upload a completed version of your questionnaire as Supporting Information when you resubmit your manuscript.

We have completed and added the questionnaire.

**3.** In your manuscript, please provide additional information regarding the specimens used in your study. Ensure that you have reported human remain specimen numbers and complete repository information, including museum name and geographic location.

If permits were required, please ensure that you have provided details for all permits that were obtained, including the full name of the issuing authority, and add the following statement:

'All necessary permits were obtained for the described study, which complied with all relevant regulations.'

If no permits were required, please include the following statement:

'No permits were required for the described study, which complied with all relevant regulations.'

For more information on PLOS One's requirements for paleontology and archeology research, see <https://journals.plos.org/plosone/s/submission-guidelines#loc-paleontology-and-archaeology-research>.

We have added to the Data and Code availability section the following statement: [page 45]

- All necessary permits were obtained for the described study, which complied with all relevant regulations.

**4.** Thank you for stating the following in the Acknowledgments Section of your manuscript:

“This research was funded by the London Arts & Humanities Partnership (LAHP), the UCL Institute of Archaeology, the Ministry of Education of the Republic of Azerbaijan and the National Environmental Isotope Facility (NEIF Grant 2777).”

We note that you have provided funding information that is currently declared in your Funding Statement. However, funding information should not appear in the Acknowledgments section or other areas of your manuscript. We will only publish funding information present in the Funding Statement section of the online submission form.

Please remove any funding-related text from the manuscript and let us know how you would like to update your Funding Statement. Currently, your Funding Statement reads as follows:

“This research was funded by the London Arts & Humanities Partnership (LAHP) (Authors: GM), the UCL Institute of Archaeology Small Research Grant (Authors: GM), the Ministry of Education of the Republic of Azerbaijan (Authors: NI) and the National Environmental Isotope Facility (NEIF Grant 2777) (Authors: GM & NI)

<https://www.lahp.ac.uk/>

<https://edu.gov.az/en/>

<https://www.isotopesuk.org/>

<https://www.ucl.ac.uk/archaeology/>

The sponsors or funders did not play any role in the study design, data collection and analysis, decision to publish, or preparation of the manuscript”

Please include your amended statements within your cover letter; we will change the online submission form on your behalf.

Thank you for letting us know. We have added the amended statement to the cover letter. We have now removed it from the manuscript main text.

“This research was funded by the London Arts & Humanities Partnership (LAHP) (Authors: GM), the UCL Institute of Archaeology Small Research Grant (Authors: GM), the Ministry of Education of the Republic of Azerbaijan (Authors: NI) and the National Environmental Isotope Facility (NEIF Grant 2777) (Authors: GM & NI)

<https://www.lahp.ac.uk/>

<https://edu.gov.az/en/>

<https://www.isotopesuk.org/>

<https://www.ucl.ac.uk/archaeology/>

The sponsors or funders did not play any role in the study design, data collection and analysis, decision to publish, or preparation of the manuscript”

**5.** When completing the data availability statement of the submission form, you indicated that you will make your data available on acceptance. We strongly recommend all authors decide on a data sharing plan before acceptance, as the process can be lengthy and hold up publication timelines. Please note that, though access restrictions are acceptable now, your entire data will need to be made freely accessible if your manuscript is accepted for publication. This policy applies to all data except where public deposition would breach compliance with the protocol approved by your research ethics board. If you are unable to adhere to our open data policy, please kindly revise your statement to explain your reasoning and we will seek the editor's input on an exemption. Please be assured that, once you have provided your new statement, the assessment of your exemption will not hold up the peer review process.

The data is now accessible on Mendeley: <https://data.mendeley.com/preview/24dyjf7c5j?a=30f08d2e-3750-4742-bf01-3c54f3cd36a6>

**6.** We note that you have referenced **(Batiuk, S. Migration Theory and the Distribution of the Early Transcaucasian Culture. Unpublished PhD dissertation, University of Toronto (2005**) and **(Longford, C. Plant Economy of the Kura-Araxes: A Comparative Analysis of Agriculture in the Near East from the Chalcolithic to the Middle Bronze Age. Vol. 1 & 2. Unpublished PhD thesis, University of Sheffield, Sheffield (2015))** which has currently not yet been accepted for publication. Please remove this from your References and amend this to state in the body of your manuscript: (ie “Bewick et al. [Unpublished]”) as detailed online in our guide for authors

<http://journals.plos.org/plosone/s/submission-guidelines#loc-reference-style>

They have both been removed from the in text references and bibliography.

**7.** We note that Figures 1, 3 and 4 in your submission contain [map/satellite] images which may be copyrighted. All PLOS content is published under the Creative Commons Attribution License (CC BY 4.0), which means that the manuscript, images, and Supporting Information files will be freely available online, and any third party is permitted to access, download, copy, distribute, and use these materials in any way, even commercially, with proper attribution. For these reasons, we cannot publish previously copyrighted maps or satellite images created using proprietary data, such as Google software (Google Maps, Street View, and Earth). For more information, see our copyright guidelines: <http://journals.plos.org/plosone/s/licenses-and-copyright>.

We require you to either (1) present written permission from the copyright holder to publish these figures specifically under the CC BY 4.0 license, or (2) remove the figures from your submission:

**1. You may seek permission from the original copyright holder of Figures 1, 3 and 4 to publish the content specifically under the CC BY 4.0 license.**

**We recommend that you contact the original copyright holder with the Content Permission Form** (http://journals.plos.org/plosone/s/file?id=7c09/content-permission-form.pdf) and the following text:

“I request permission for the open-access journal PLOS ONE to publish XXX under the Creative Commons Attribution License (CCAL) CC BY 4.0 (http://creativecommons.org/licenses/by/4.0/). Please be aware that this license allows unrestricted use and distribution, even commercially, by third parties. Please reply and provide explicit written permission to publish XXX under a CC BY license and complete the attached form.”

**Please upload the completed Content Permission Form or other proof of granted permissions as an "Other" file with your submission.**

**In the figure caption of the copyrighted figure, please include the following text: “Reprinted from [ref] under a CC BY license, with permission from [name of publisher], original copyright [original copyright year].”**

2. If you are unable to obtain permission from the original copyright holder to publish these figures under the CC BY 4.0 license or if the copyright holder’s requirements are incompatible with the CC BY 4.0 license, please either i) remove the figure or ii) supply a replacement figure that complies with the CC BY 4.0 license. Please check copyright information on all replacement figures and update the figure caption with source information. If applicable, please specify in the figure caption text when a figure is similar but not identical to the original image and is therefore for illustrative purposes only.

The following resources for replacing copyrighted map figures may be helpful:

USGS National Map Viewer (public domain): <http://viewer.nationalmap.gov/viewer/>

The Gateway to Astronaut Photography of Earth (public domain): <http://eol.jsc.nasa.gov/sseop/clickmap/>

Maps at the CIA (public domain): <https://www.cia.gov/library/publications/the-world-factbook/index.html> and <https://www.cia.gov/library/publications/cia-maps-publications/index.html>

NASA Earth Observatory (public domain): <http://earthobservatory.nasa.gov/>

Landsat: <http://landsat.visibleearth.nasa.gov/>

USGS EROS (Earth Resources Observatory and Science (EROS) Center) (public domain): <http://eros.usgs.gov/#>

Natural Earth (public domain): <http://www.naturalearthdata.com/>

Thank you for your message regarding Figures 1, 3, and 4.

We have reviewed all figures to ensure full compliance with the CC BY 4.0 license.

- **Figure 3 (now Figure 1):** Location and elevation map of Maxta I, created using data from the *ASTER Global Digital Elevation Model (GDEM) Version 3*, a public NASA/METI product available via [NASA Earthdata](https://www.earthdata.nasa.gov/). Data processed in ArcMap v10.8.2.
- **Figure 4 (now Figure 2):** Spatial variation of temperature, precipitation, and snow cover across the Caucasus, based on open-access NASA datasets from [Giovanni, NASA GES DISC](https://giovanni.gsfc.nasa.gov/giovanni/), processed in ArcMap v10.8.2 and assembled using [BioRender.com](https://app.biorender.com/).
  Both datasets are distributed under NASA’s Open Data and Information Policy, which permits unrestricted reuse with attribution.

To comply fully with copyright requirements, we have **removed Figure 1** (originally based on an Esri basemap).

**8.** We note that there is identifying data in the Supporting Information file < S5_Selected samples isotope analysis.xlsx and S7_collagen isotope analysis.xlsx >. **Due to the inclusion of these potentially identifying data, we have removed this file from your file inventory.** Prior to sharing human research participant data, authors should consult with an ethics committee to ensure data are shared in accordance with participant consent and all applicable local laws.

Data sharing should never compromise participant privacy. It is therefore not appropriate to publicly share personally identifiable data on human research participants. The following are examples of data that should not be shared:

-Name, initials, physical address

-Ages more specific than whole numbers

-Internet protocol (IP) address

-Specific dates (birth dates, death dates, examination dates, etc.)

-Contact information such as phone number or email address

-Location data

-ID numbers that seem specific (long numbers, include initials, titled “Hospital ID”) rather than random (small numbers in numerical order)

Data that are not directly identifying may also be inappropriate to share, as in combination they can become identifying. For example, data collected from a small group of participants, vulnerable populations, or private groups should not be shared if they involve indirect identifiers (such as sex, ethnicity, location, etc.) that may risk the identification of study participants.

Additional guidance on preparing raw data for publication can be found in our Data Policy (<https://journals.plos.org/plosone/s/data-availability#loc-human-research-participant-data-and-other-sensitive-data>) and in the following article: <http://www.bmj.com/content/340/bmj.c181.long>.

Please remove or anonymize all personal information, ensure that the data shared are in accordance with participant consent, and re-upload a fully anonymized data set. Please note that spreadsheet columns with personal information must be removed and not hidden as all hidden columns will appear in the published file.

Thank you. This is data from archaeological sheep and goat specimens not from humans.

**9. Please include captions for your Supporting Information files at the end of your manuscript, and update any in-text citations to match accordingly. Please see our Supporting Information guidelines for more information**: <http://journals.plos.org/plosone/s/supporting-information>.

If the reviewer comments include a recommendation to cite specific previously published works, please review and evaluate these publications to determine whether they are relevant and should be cited. There is no requirement to cite these works unless the editor has indicated otherwise.

We have now added captions for our Supporting Information files at the end of the manuscript as well as updated any in-text citations.

**Reviewers' comments:**
 **Reviewer #1:**

This manuscript provides a comprehensive analysis of seasonal pastoral strategies employed by the population of Maxta I ascribed to the Kura-Araxes culture in Nakhchivan, Azerbaijan, southern Caucasus. The proposed multi-method approach included an extensive program of studies based on a variety of methods employed to identify seasonal mobility of the herders related to development of upland herding. The comparison of pastoral strategies noted for their diversity provides an opportunity to discuss economic activity, temporal and spatial organization of the Maxta I occupants’ settlement strategy, assess the level of adaptation to local landscapes, exploited resources as well as seasonal mobility within the exploited lowland–upland pastures.

Based on the case study of a lowland settlement, the authors use the seasonality/mobility model they have developed to reconstruct one of the most important components of the Kura-Araxes subsistence model based on the agro-pastoral system, provide evidence supporting use of diverse strategies in a rather large region occupied by this culture across southwest Asia and suggest that this economic potential also existed in other Kura-Araxes areas. This model is based on livestock management when a greater part of caprines is seasonally moved between the upland and lowland pastures while a smaller part is raised at the site or nearby. The authors note dominance of sheep in the faunal assemblage compared to goats, almost complete absence of suids, and a rather high percentage of wild animal bones. This paper is the first attempt to analyze this dual economic model for the Kura-Araxes culture when some residents remained at the settlement year-round engaging in various household activities while other occupants moved herds to nearby and distant pastures.
There is no doubt that this study is exciting as it shows advantages of comprehensive approaches in the analyses of archaezoological material making it possible to discuss economic strategies in prehistory. The paper can be recommended for publication in PLOS ONE. However, it needs some clarifications that will reinforce main conclusions derived by the authors.

We thank the reviewer for their careful assessment of our manuscript and for recognising its contribution to the study of Kura-Araxes subsistence strategies and pastoral mobility. We appreciate the constructive suggestions provided, which have helped us to refine our arguments and improve the clarity of our presentation. In the revised version, we have addressed each of the reviewer’s comments in detail.

**1.** It is better to provide the results of 14C dating available for the site structures analyzed, at least in the Suppl. Data. Figure 2 that the authors make reference to (pg. 5) does not contain any dating results.

Many thanks for this suggestion. We are currently dating the three structures and the samples with associated isotopic/zooms evidence. However, as this is a large separate study it will be published independently at a later stage of the project. We are therefore referring to the previous dates of Maxta from Ristvet at al. 2019.

**2.** The authors should provide stronger arguments in support of such sector as wool production. The authors use the estimated mortality rates of animals (sheep and goats) as the main evidence of wool production and say that the animal slaughter age can point to such secondary products as milk and wool. Examples of using both plant (made from flax) and wool textiles in the southern Caucasus, at least, in contemporaneous cultures where such textiles have been identified should be provided (see in Ananauri big kurgan № 3. Tbilisi: Georgian National Museum/ 2016; Bedianashvili, G., Jamieson, A., Longford, C., Martkoplishvili, I., Paul, J., Sagona, C., 2022. Evidence for textile production in Rabati, Georgia, during the Bedeni phase of the Early Kurgan period. Journal of Archaeological Sciences: Reports 43, 103467. ISSN 2352-409X). More importantly, the recent studies of wool textile from the southern Caucasus have shown that it is made of goat underwool (Archaeological Research in Asia 43 (2025) 100635). The results of the study are well correlated with the data from Arslan-Tepe.

We thank the reviewer for this insightful comment. We agree that our discussion of wool production requires additional support. In the revised manuscript, we have taken the following steps:

1. Expanded the argument beyond mortality profiles. We now clarify the limitations of using slaughter age as the sole indicator of secondary product exploitation. To make this point explicit, the revised passage (page 38, line 895) reads:


    “*However, slaughter profiles on their own cannot provide sufficient grounds for inferring a focus on secondary products.*”
2. Incorporated comparative evidence from Bronze Age contexts in the southern Caucasus. We now reference archaeological evidence for both flax and wool/hair textiles from contemporaneous sites:

Bedianashvili, G., Jamieson, A., Longford, C., Martkoplishvili, I., Paul, J., & Sagona, C. (2022). Evidence for textile production in Rabati, Georgia, during the Bedeni phase of the Early Kurgan period. *Journal of Archaeological Science: Reports*, 43, 103467.

Kvavadze, E. (2016). *Palynological study of organic remains from the Ananauri Kurgan № 3*. In *Ananauri Big Kurgan № 3* (pp. 156–172). Tbilisi: Georgian National Museum.

3. Addressed recent studies on fiber origin. We added reference to the recent work showing that Bronze Age wool textiles in the Caucasus were manufactured from goat underwool, aligning well with the evidence from Arslantepe:

Shishlina, N.I. & Chernova, O.F. (2025). Bronze Age wool textile and fur items from northern Eurasia: Identification of the fiber origin and differentiation between domestic animal species. *Archaeological Research in Asia*, 43, 100635.

Accordingly, the revised passage in the manuscript (page 38, line 60) now reads:

*“Comparative evidence from Bronze age contexts in the southern Caucasus likewise points to the exploitation of both plant- and wool or goat hair-based textiles[248-250].”*

Using the results of tracewear analysis of similar items from other Bronze Age sites, and judging by the form of the horse ulnar bones presented in Fig. 2 (c) and in the Supplementary file, these ulnar bones could not be used in textile production. Most likely, these items are piercers or perforators similar to tools used in leather production that, presumably, was one of main productions at the settlement.

We thank the reviewer for this important comment. We acknowledge that ulna tools were highly multifunctional and cannot be securely assigned to a single activity in the absence of direct tracewear analysis from Maxta. As demonstrated by Keddie (2010), ulna bones of different taxa (sheep/goat, deer, horse etc) were employed in a range of tasks, including piercing hides, pressing fibres in basketry, splitting bark, and processing fish. While textile production remains one possible use, we agree that the Maxta specimens are unlikely to have been used exclusively for that purpose. In light of the reviewer’s observation, we now stress in the revised manuscript that these tools are more plausibly understood within a broader spectrum of functions, with leatherworking representing a particularly likely activity at the settlement. The revised passage (page 39, line 865) now reads:

*“Although textile production cannot be excluded as a possible function, ulna bone tools can be employed in a wide range of activities, including hide piercing, fibre pressing, bark splitting, and fish processing[251]. In this light, the Maxta ulna awl specimens are best understood as multipurpose implements, with leather and fibre working whether plant- or animal-based among several plausible associated uses. Comparable awls are also attested at other KAC sites, in eastern Azerbaijan, Iran[252].”*

We also adjusted the Figure 2c caption to “Evidence for possible leather and fiber (animal and/or plant) working” (page 8).

Reference added:

Keddie, G. (2010). Ulna bone tools: Identifying their function. *The Midden*, 44(3/4), 23–28

Decaix, Alexia, Fatemeh Azadeh Mohaseb, Sepideh Maziar, Marjan Mashkour, and Margareta Tengberg. 2020. “Subsistence Economy in Kohneh Pasgah Tepesi (Eastern Azerbaijan, Iran) during the Late Chalcolithic and the Early Bronze Age Based on the Faunal and Botanical Remains.” In *The Iranian Plateau during the Bronze Age: Development of Urbanisation, Production and Trade*, edited by Jan-Waalke Meyer, Emmanuelle Vila, Marjan Mashkour, Michèle Casanova, and Régis Vallet, 75-88. Lyon: MOM Éditions

It would be interesting to know if there are textile impressions on clay vessels or coating on the dwellings.

Many thanks for your comment. There are no impressions of textiles on any clay vessels or dwellings from Maxta unfortunately.

**3.** The taphonomic analyses of animal bones are not very clear. Are there cases when the anatomical bone composition of a specific animal species includes all elements of the skeleton? If there are, it may suggest that animal carcasses were cut into pieces at the site. First of all, it holds true for cattle. Presence of bones of newly born animals of these species may also indicate that cattle was not only kept but also raised at the site. Please clarify if such data are available.

We thank the reviewer for highlighting this point. To clarify, we have added an analysis of cattle and caprine body part representation to the supplementary material (S5 Dataset: Body part representation). Cattle remains are dominated by head, shoulder and haunch. All anatomical elements are present on site apart from the lower fore-limb (ulna and radius) and part of the lower hind-limb (tibia). Caprine elements remain generally poorly represented except for mandibles and humeri (S5). This profile suggests caprine butchery is carried out locally on the household level, while most processing and consumption appears to take place elsewhere. Whether this relates to the use of space, systems of provisioning or patterns of mobility needs further investigation. Cattle are also primarily butchered on the household level, with meat rich parts well represented, suggesting the processing or discard of unfavourable parts elsewhere.

We have added additional dental wear data for cattle (small sample; S5 Dataset, Table I). The mortality profiles based on dental wear (however we are dealing with small sample sizes), also indicate that most cattle are culled young, between the ages of 0 and 28 months. Only age classes B-D are present. The presence of age class B suggests the local breeding of cattle at Maxta I. The fusion profile shows there is a presence of very young cattle at Maxta I, younger than 7-10months of age (Stage A). However, the profile is aberrant, possibly caused by the small sample size in stage A. The most significant culling of animals takes place between stage C and D. The great majority of animals do not survive past the final fusion stage D (42-48+ months).

We have clarified these aspect by adding the following to the results (page 28, line 606) :

*“Cattle mortality profiles, based on small fusion and dental wear samples, indicate that most individuals were culled between 0–28 months (Classes B–D); a few very young animals (<10 months, Stage A) are present but may be biased by sample size, and few survived beyond the final fusion stage (>42–48 months) (Supplementary material 3).”*

In the discussion (page 35, lines 787) :

*“Furthermore, cattle seem to have been locally bred (class B), but the overall narrow age range is consistent with a seasonal management model.”*

We also added an additional section in the results on the body part analysis (page 27, line 565) :

***“****The anatomical profile of caprine remains is less complete and dominated by mandibles and humeri, whereas cattle are represented mainly by head, shoulder, and haunch elements, with nearly all skeletal parts present except for sections of the lower limbs (Table E in S5 Dataset).”*

**4.** Probably, the authors of this study did some experimenting cut of grass in summer pastures (plots) located around the site to determine percentages of C3 and C4 plants in such pastures. Such data, if available, could reinforce the suggestions made by the authors regarding use of summer pastures with pure C3 vegetation located far from the site.

We thank the reviewer for this suggestion. In our study, we did not perform new vegetation cutting experiments; instead, we compiled published δ¹³C values of C₃ and C₄ plants from the Caucasus to represent the isotopic range of local vegetation. These values were used to establish regional cut-offs for pure C₃ (–11.58 ± 3.00 ‰) and pure C₄ (4.18 ± 1.17 ‰) diets in enamel (see p. 24, line 495) of the revised manuscript; Table B in S4 Dataset). Modern plant δ¹³C values were corrected by +1.5 ‰ to account for the Suess (fossil fuel) effect. We believe this regional dataset provides a robust framework for interpreting the enamel δ¹³C values presented in our study.

**5.** Maybe, it will be useful to indicate what trees and shrubs grow around the site as the authors make reference to browsers which fed on leaves and shoots in winter. Such data, certainly, cannot be used as direct evidence of the landscape in prehistory. However, the authors argue that the climate at the time of Maxta I was similar to modern climate; therefore, this information can help get an insight in core fodder resources in winter at the time of the site occupation.

We thank the reviewer for this helpful suggestion. In the revised manuscript we now refer to S2 Dataset, which provides a list and distribution of *C₃* and *C_4_* plant species in the Caucasus, together with a vegetation map and carbon isotope values of representative species. In addition, we have clarified this in text on page 10, line 196 :

“*The regional vegetation reflects a dynamic mosaic of C₃ and C₄ plant communities (Table A in S4). C₃ grasses such as Festuca, Stipa, and Agropyrum dominate in spring and early summer but senesce during the hot, dry season. In contrast, C₄ taxa expand in late summer and autumn, including annual grasses (Bothriochloa) and halophytic shrubs. Genera such as Halostachys and Kalidium are predominantly C₄, while Salsola, Suaeda, and Halocnemum include both C₃ and C₄ species. These woody halophytes often remain green into winter and, together with hardy C₃ shrubs like Capparis spinosa and Artemisia fragrans, would have provided dependable browse when C₃ grasses were no longer available. Moreover, local microclimatic conditions, such as saline flats, disturbed soils, and sheltered south-facing slopes, support persistent C₄ plant growth even into cooler seasons in the Sharur plain[92,93]. C₄ grasslands have also been documented in northwestern Iran until late autumn[94].δ¹³C values from modern vegetation in the region also exhibit elevation-linked enrichment****^63^****. Available palaeoclimatic evidence suggests that Early Bronze Age climate conditions in the region were broadly comparable to those of today[95,96]. Full details on regional climate, vegetation, hydrology, and isotopic variation are provided in S1 File, S3 and S4 Dataset. “*

**6.** Probably, it should be indicated that the snowfree period (according to modern climatic data with which the authors correlate presumed local climatic characteristics during the period when the site was occupied) lasted almost 11 months while the average snow cover does not exceed 25 mm; for this reason, practically all local pastures around the settlement can be used during the cold time of the year.

We thank the reviewer for this helpful suggestion. We have added information on snow cover and pasture availability to clarify the climatic context of the Sharur Plain. The following sentence has been added to the environmental background section: page 9, line 184:

*“ The Sharur Plain is snow-free for c. 11 months per year, with average snow cover not exceeding 25 mm, indicating that local pastures remain accessible year-round (Fig 3). ”*

**7.** It is not quite clear if there are differences in the nitrogen and carbon isotopic composition of the animals that grazed on the manured pastures around the settlement and those that were moved to more extensive upland pastures; the data on the δ15N values of the analyzed animal sample are similar. And while the authors note that there is no direct evidence of haymaking, was the use of straw possible? For example, are there any impressions of straw on the walls and the floor of the mud-brick and wattle-and-daub dwellings?

We thank the reviewer for this thoughtful comment. For the collagen isotope analysis, we focused on mandibles that also have sequential enamel isotope data, as these provide direct evidence of seasonal mobility. A small number of additional specimens were analysed; however, because these lack associated sequential data, their specific pastoral practices cannot be securely identified. To make this distinction clearer, we have added a column in S6 Dataset, Table A, indicating which specimens are linked to sequential isotope series.We have also clarified this point in the main text (p. 33, line 738):

*“Mandibles selected for collagen isotope analysis were those with associated sequential enamel isotope records, allowing us to link seasonal practices directly to collagen δ¹³C and δ¹⁵N values. Additional specimens lacking sequential data were analysed but cannot be securely assigned to seasonal strategies.”*

Regarding the possibility of straw use, no straw temper has been identified in the ceramic fabric. However, traces of straw can be observed in the composition of the mudbricks used in house construction. We have clarified this in the revised discussion (p. 32, line 703):

“*Summer-harvested C₄ grasses may have been stored and fed to animals during winter, although there is no direct evidence for this practice.However, traces of hay or straw have been identified in the composition of mudbricks and wattle and daub used in house construction[84]”*

**8.** Reference to the archaeobotanical data obtained from the settlement integrated into the analyses (line 648) is absent, though it is made below (line 763). As the authors propose the seasonal adaptive agro-pastoral model, the available archaeobotanical data (wheat, barley, fruits as indicated in the model in Figure 8) should be provided in the text. Have any straw impressions been documented on the mud-brick and wattle-and-daub dwellings? Such data can imply that winter fodder (straw) was hoarded.

We thank the reviewer for drawing attention to this. We have now integrated the published archaeobotanical results from Maxta I into the discussion of the seasonal agro-pastoral model (see p. 40, line 896). We have also removed fruits and legumes from Figure 7, as these categories are not supported by the available archaeobotanical evidence.

*“Archaeobotanical evidence[66] from Maxta I is based on a small, spatially restricted sample and should be regarded as preliminary. The assemblage is dominated by barley (Hordeum vulgare), with minor bread/macaroni wheat (Triticum aestivum/durum), small quantities of wild and weedy taxa (e.g., Boraginaceae, Galium), and only limited evidence for the pulse bitter vetch (Vicia ervilia); no C₄ crops have been recovered. Barley was probably autumn-sown[257], a practice that could have benefited from winter dung deposition. Together with the isotopic results, this pattern is consistent with a possible synchronization of pastoral and agricultural cycles: caprines may have been moved to higher pastures after the spring growing season and early summer harvest, when lowland fields had dried to stubble and fresh forage was available in the highlands, and returned in autumn to fertilise fields. However, this scenario remains tentative and requires confirmation through broader archaeobotanical and plant isotopic evidence.”*

Regarding the possibility of straw use, no straw temper has been identified in the ceramic fabric. However, traces of straw can be observed in the composition of the mudbricks used in house construction. We have clarified this in the revised discussion (p. 32, line 703):

“*Summer-harvested C₄ grasses may have been stored and fed to animals during winter, although there is no direct evidence for this practice.However, traces of hay or straw have been identified in the composition of mudbricks and wattle and daub used in house construction[84].”*

**9.** The seasonality of the settlement is based on the modelling of δ18O sequences and variations in the δ13С values in the intra-tooth samples, seasonality of birth, absence of newly born caprines among the settlement animals (which means lambing/kidding outside the settlement in the spring and autumn for sheep and late autumn for goats), as well as variations in the collagen stable δ13С and δ15N values in the animal bones.

According to the authors, they plan to analyze variations in the strontium isotopic ratios which will confirm one of the conclusions on lambing and kidding outside the settlement in the summer and autumn pastures.

The authors should also note that there is one more method that can be used to confirm or reject the conclusions regarding year-round or seasonal grazing of various species of animals in the pastures, it is the dentum analysis of incremental bands in the cementum and detrine of teeth (Klevezal G.A. Recording structures of mammals. Determination of age and reconstruction of life history. Rotterdam: Balkema Publish. House. 1996). This method is employed to determine the season of animal slaughter and thereby confirm the seasonal pastoral model under discussion.

We thank the reviewer for this valuable suggestion. We agree that incremental band analysis of tooth cementum (Klevezal 1996) provides an additional independent approach for determining season of death and thus testing models of seasonal grazing and mobility. While our current study focuses on sequential enamel δ¹⁸O/δ¹³C modelling and bone collagen isotopes, we have now added a note in the discussion acknowledging the potential of cementum analysis for refining seasonal herding reconstructions and plan to consider this method in future work (see p.43, line 970):

*“Future work could also apply incremental growth band analysis of tooth cementum [273] to determine the season of slaughter and thereby provide an independent test of the seasonal herding model proposed here.”*

**10.** The paper discussed focuses on the analysis of sheep/goats; however, the cattle is the second largest share of the reconstructed herd of the local population. Apparently, cattle played an important role in secondary productions, especially, milking, dung collection (used as fuel and fertilizer for fields), traction as well as meat production. The role of such animals in the discussed seasonal agro-pastoral model should be assessed, e.g. was all cattle raised at the site? Was the cattle kept in the stalls in winter and did it graze on pastures around the settlement in summer, how far could such pastures be located?

We thank the reviewer for highlighting the importance of cattle within the Maxta herd and their likely contribution to secondary products and field management. Our study focuses on sheep and goats because limited ageing and no isotopic data are currently available for cattle beyond species counts. We have now clarified this limitation in the discussion and added a note on the potential role of cattle within the agro-pastoral system, emphasising the need for future analyses (see p. 43, line 987).

“*Although the present study focuses on sheep and goats, cattle constituted a substantial part of the reconstructed herd and likely contributed to secondary products such as milk, dung (used as fuel and manure), traction, and meat. However, current evidence for cattle is restricted to species counts, with limited ageing and no isotopic data available to reconstruct their seasonal management. Future analyses will be essential to clarify the role of cattle within the seasonal agro-pastoral system proposed here. “*

**10.** The authors should carefully check the numbering of the references in the text and the references list. See, for example, line 795: In the References section Frachetti paper is with the number 199, but in the text it is numbered 200.

Thank you for altering us. We have carefully checked the references and revised the numbering.

**Reviewer #2:**

This paper mainly presents archaeozoological and isotopic results that helps describing the agro-pastoral strategies of the Kura-Araxes inhabitants of Maxta.

**1.** The main problem of the paper is the over interpretation of the data. And also, the tendency to inflate the impact of the study is clumsy. It is a decent study but it is not establishing a strong model to understand the diversity of pastoral strategies in SW Asia. Azerbaijan is a small territory and Nakhchivan even more. It is home to a dozen of archeological excavations but of course it is necessary to see the image from the whole region as there are interesting projects in Armenia and Georgia as well. Therefore a sentence such as *“Maxta I is among the few systematically excavated Kura-Araxes Culture (KAC) sites in Nakhchivan, and the only one in Azerbaijan where pastoral mobility and economic organisation are investigated in depth”* sounds clumsy.

We thank Reviewer #2 for this thoughtful comment. We agree that our original phrasing could be read as overstating the significance of Maxta I for understanding pastoral strategies across the entire Southwest Asian Kura–Araxes sphere. Our intention was simply to emphasise that Maxta I provides the first in-depth analysis of KAC pastoral mobility and economic organisation in Azerbaijan, and that the combination of traditional zooarchaeology with biomolecular methods used here offers a framework that can be applied to other Kura–Araxes sites.

To clarify this point, we have revised the relevant passage (p. 7, line 133) to read:

“ *The results from Maxta I contribute to a comparative framework applicable across Southwest Asia, offering a localised perspective that complements evidence from other regions of the South Caucasus; including Armenia[54-59], Georgia[16,60,61], and northwestern Iran[63], to advance a more comprehensive understanding of pastoral strategies.”*

We also thank the reviewer for highlighting the need to situate Maxta I within the broader regional context. We fully agree and already cite all published studies known to us from Armenia, Georgia, and Iran. To strengthen this point, we have now added additional references from Georgia and Armenia) (p. 6, line 108):

“*Sequential stable isotope analysis of herbivore enamel has been performed in Turkey[53], Armenia[54-59], Georgia[16, 60,61], the North Caucasus[62], and Iran[63]. Within the Kura-Araxes Culture, such studies are increasing but remain comparatively few, and each new dataset contributes to refining our understanding of herding and mobility in the region.”*

2. The paper should be refocused on the produced data and their interpretation. Part of the text and the supplementary data are not fully relevant to the aim of the paper. For these reasons I recommend a major revision.

We thank the reviewer for this overall assessment. We have carefully revised the manuscript to address each of the reviewer’s points and to ensure that the paper is now clearly focused on the produced data and their interpretation.

3. There is an impressive list of 282 references. It’s too much. It is not necessary to show that you have read everything. This is not a review article. It is necessary to be more synthetic. Furthermore, in the introduction to the Kura-Araxes culture, there is no hierarchy in the sources. Everything is cited from recognized syntheses to anecdotical papers. Some interesting references are hsowever missing. You don’t mention the fierce debate about the origins of the Kura-Araxes that concerns evidence from Nakhchivan (Marro et al. (2014) On the Genesis of the Kura-Araxes Phenomenon: New Evidence from Nakhchivan (Azerbaijan), with reply from Palumbi and Chataigner, and reply of Marro et al). You do not either discuss the place or specificities of Maxta in comparison to other KA settlements excavated in Nakhchivan or in the region.

We thank the reviewer for this helpful observation. We have reduced the overall number of references in the background. However, we cannot remove references from the methodology which are substantial due to the various methods deployed. We are also reluctant to remove local azeri references. We have however added the missing discussion of the debate on the origins of the Kura–Araxes culture, including the evidence from Nakhchivan and the exchange between Marro et al. (2014) and Palumbi & Chataigner (p. :3, line 47).

*“The origins of the Kura–Araxes phenomenon remain debated, with evidence from Nakhchivan playing a central role (Marro et al. 2014; Palumbi & Chataigner 2014; Marro et al. 2015).”*

In addition, we have briefly situated Maxta I in relation to other excavated Kura–Araxes sites in Nakhchivan and the South Caucasus to acknowledge its regional context. A full comparative analysis of Maxta I with these sites would be valuable but is beyond the scope of this article, which focuses on presenting new zooarchaeological and isotopic data.

Page 8, line 159:

“Maxta I can therefore be considered in relation to these and other excavated Kura–Araxes settlements in Nakhchivan[74-77](Fig 1).”

**4.** Line 139. I don’t see the link between Figure 2 and the fact that bones from Maxta are currently radiocarbon dated.

We thank the reviewer and have now removed the reference to Figure 2.

**5.** Figure 3 could be completed with the hydrological system (main rivers and lakes), the name of the countries and maybe a few modern cities to help the reader.

We thank the reviewer for this valuable suggestion. We have added a little side regional map to orient the reader. We have also added additional Kura-Araxes settlements in Nakhchivan, Armenia, and Iran to further improve geographic context. We have also indicated the Lesser Caucasus range. We have chosen not to include the hydrological system in this figure, as doing so would significantly clutter the map. However, a detailed hydrological representation of the site and surrounding region is provided in Figure A in S3 Dataset.

**6.** Figure 4. Is not very useful in the text but called many times in the supplementary document, it should be moved there.
When you mention modern δ18O values, in the supplementary for instance, you use modeled values (from Bowen & Revenaugh) but the way you present them isn’t clear, it looks like you refer to measured values, it is different. Line 177 for example. Aren't there actual values from the GNIP in the area ?

We appreciate the reviewer’s comment. However, we would prefer to keep Figure 4 in the main text, as another reviewer highlighted its value in providing a clear overview of the modern climatic variability. We believe that retaining it in the main text helps readers better understand the seasonal context and the comparison with our proxy data, which is central to our interpretation.

We have also clarified in the text and in the supplementary that the modern δ¹⁸O values refer to modeled precipitation values from Bowen & Revenaugh (2003) rather than measured data. At present, no GNIP stations within the immediate study area provide sufficient coverage for reliable local δ¹⁸O averages. The supplementary also contains a detailed list of all published modern δ¹⁸O values of regional water sources.

We have revised the relevant passage (p. 9, line 194) to read:

*“Modern modelled δ¹⁸O values in precipitation at Maxta I range from –15.4‰ in winter to –1.8‰ in summer[90]“*

**7.** Line 186. many publications report paleoenvironmental data from the Caucasus to investigate the climatic and environmental conditions in the past. References 105 and 106 are not relevant.

We thank the reviewer for pointing this out. We have revised the sentence and replaced references 105 and 106 with more relevant sources that specifically address palaeoclimatic and palaeoenvironmental data from the Caucasus region.

References:

Bliedtner, M., Zech, R., Zech, J., Schäfer, I. and von Suchodoletz, H., 2020. A first Holocene leaf wax isotope‐based paleoclimate record from the semi‐humid to semi‐arid south‐eastern Caucasian lowlands. *Journal of Quaternary Science*, *35*(5), pp.625-633.

Cromartie, A., Blanchet, C., Barhoumi, C., Messager, E., Peyron, O., Ollivier, V., Sabatier, P., Etienne, D., Karakhanyan, A., Khatchadourian, L. and Smith, A.T., 2020. The vegetation, climate, and fire history of a mountain steppe: A Holocene reconstruction from the South Caucasus, Shenkani, Armenia. *Quaternary Science Reviews*, *246*, p.106485.

**8.** Line 233. It would be interesting to know what the sub-set represents in % of the whole assemblage. How the subset was selected?

We thank the reviewer for this comment. We sampled approximately 25% of the faunal material from each excavated context at Maxta I. The sub-set was selected to include diagnostic postcranial elements suitable for taxonomic, metric, and taphonomic analysis, while highly fragmented or non-diagnostic specimens were excluded.

We have revised the text to (p. 12, line 260) to read:

*“ The analysed postcranial assemblage includes approximately 25% of the faunal material from each context, focusing on diagnostic elements.”*

**9.** Line 287-290. I think that it would be worth to move supplementary 8 into the main text. It is very relevant for the discussion

We thank the reviewer for this helpful suggestion. We have moved Supplementary 8 into the main text which is now Table 1 and Table 2.

**10.** Line 435-47. The collected data are not relevant due to the variability of the sampling location, and the number of values for C3 plants, to build a regional threshold. You should first explain why the common thresholds used in previous articles reporting isotopic data, needed to be redefined, if this is really the case. Line 446 you mention -4.8‰ for pure C4 but 4.8 in the supplementary data.

We thank the reviewer for this thoughtful comment. We have clarified that our goal was not to redefine existing isotopic thresholds, but to apply regionally appropriate and taxonomically representative comparative data that better reflect the environmental and ecological conditions of Maxta I.

Our C₃ plant baseline is derived from empirically measured δ¹³C values across two well-documented semi-arid steppe regions:
 (1) the Salmas plain in north-western Iran, as reported by *Samei et al.* (2023), where Köhne Shahar lies within a semi-arid foothill-steppe environment (~300–350 mm annual precipitation, open grass–shrub vegetation); and
 (2) the North Caucasus and Caspian lowlands, as described by *Shishlina et al.* (2018), representing semi-arid to arid lowland steppe zones (<300 mm rainfall) dominated by temperate grasses and chenopods.
 Together, these datasets incorporate a broader range of C₃ taxa and environmental settings than the South Caucasus plant datasets used to establish the *Janzen et al.* (2023) threshold, thereby providing a more comprehensive picture of isotopic variability within semi-arid steppe vegetation relevant to Maxta.

We fully acknowledge the −6.7 ‰ enamel cut-off for a 100 % C₃ diet proposed by *Janzen et al.* (2023), which was derived from archaeological plant δ¹³C values at Chobareti (*Messager et al.* 2015) and Mentesh Tepe (*Herrscher et al.* 2018a), together with modern vegetation data from north-eastern Iran. This cut-off represents the upper isotopic limit of **strongly water-stressed** C₃ vegetation, rather than an average regional value, and is based on a relatively limited range of taxa.

In the revised manuscript (p.24, line 504), we now explicitly reference this value as a maximum C₃ cut-off, noting that *“According to Janzen et al. (2023), synthesis of modern and archaeological plant δ¹³C data from the South Caucasus suggests that values around −6.7 ‰ in enamel represent the extreme enrichment possible for diets derived entirely from water-stressed C₃ plants.”*

Concerning the cut-off for C_4_ plants, previous studies have generally relied on global average C₄ cut-offs, whereas we consider it more appropriate to use the regionally specific dataset of *Rudov et al.* (2020), which focuses on C₄ plants in south-western Asia. This provides a better contextual match for the vegetation patterns surrounding Maxta than using a global average.

We recognise that a general limitation faced by all isotopic studies in the region is the absence of modern local plant isotope baselines. We have added a statement in the methods as well as limitation section to clarify this limitation (p. 24, line 514):

*“A limitation of this approach is the absence of modern isotopic measurements of C₃ and C₄ plants from the immediate vicinity of Maxta, which prevents the establishment of a truly local baseline. As a result, the isotopic thresholds used here rely on the closest available regional proxies and should be interpreted as approximate comparative values rather than fixed local end-members.”*

Finally, we thank the reviewer for alerting us to the typographical error concerning the δ¹³C value for pure C₄ plants. This has now been corrected to - 6.3‰ (reflecting cut-offs in bone collagen not enamel), consistent across both the main text and Supplementary Data.

**11.** ZooMs. It is difficult to understand figure 5e. You mention in the text 20 samples from ZooMs but in the figure it is written N=26

We thank the reviewer for highlighting this error and the lack of clarity in Figure 5e - now Figure 4e. The figure has now been corrected and simplified to ensure consistency between the sample sizes reported in the text and those shown in the figure.

Specifically, the ZooMS dataset now correctly shows N = 20, reflecting the total number of mandibles analysed. The figure and caption have been revised to clearly explain the relationship between the four panels:

***“e.*** *Identification of Ovis and Capra mandibles and postcranial elements using morphological and ZooMS analyses. ZooMS improved genus-level resolution of previously classified Ovis/Capra remains.The top panels show morphological identifications for all specimens (left, N = 226) and for the subset of mandibles analysed by ZooMS (right, N = 20). The bottom panels show ZooMS identifications for these 20 mandibles (right) and combined results for the entire assemblage (left, N = 226), where molecular identifications were integrated with morphological observations to refine overall taxonomic assignments. Colours indicate Ovis/Capra (purple), Ovis (blue), and Capra (teal). “*

**12.** Lines 600-619. I do not agree with the idea that pattern A.3 correspond to animals grazing in highlands in the summer. They display carbon values around -9‰. It is not low enough for the highlands. Calculation by Samei (2013) Highland pastoralism in the Early Bronze Age Kura-Araxes cultural tradition: "Stable oxygen (δ^18^O) and carbon (δ^13^C) isotope analyses of herd mobility at Köhne Shahar in northwestern Iran", and also modern transhumant sheep from Armenia (published in Janzen et al 2023 Neolithic herding practices in the Southern Caucasus: Animal management in the early 6^th^ millennium BCE at Masis Blur in Armenia’s Ararat Valley)" show that alpine meadows correspond to values around -12‰ in the enamel. The anti-phase relationship is due to a C4 enriched diet in the winter, not to highland pastures in the summer. Your main evidence is that Maxta is in an arid environment full of C4 plants. Actually, the landscape around Maxta is not arid, as it can be seen from a google earth image. The valley of the Araxes is rather marshy and it was probably even more in the past before the control of the Araxes river by dams. Arid and salty environments can be found in the area, probably more on the hilly flanks, but considering that Maxta should have a C4 signal for local grazing might be wrong.

Thank you very much for your detailed and thoughtful comments. We appreciate your insights and your references to Samei (2023) and Janzen et al. (2023), both of which we are familiar with and have cited in the manuscript.

Regarding your point on the carbon isotope values, we agree that the relationship between δ¹³C values and altitude is complex and context-dependent. While values around -12‰ have been reported for certain alpine meadow contexts, this should not be considered a universal highland cutoff across the Caucasus region. As discussed by Samei (2023), the δ¹³C variation with elevation in the Caucasus shows an increase of approximately +0.46‰ per 100 m, and grazing altitudes likely varied considerably between sites. Moreover, multiple environmental and ecological factors can influence the carbon isotope composition beyond altitude alone.

Concerning the environmental setting of the Sharur plain, we acknowledge that local conditions are heterogeneous. However, characterising it as a purely C₃ environment would not be accurate. Present-day climatic data indicate an annual precipitation of approximately 300 mm, which classifies the area as semi-arid. Such conditions support a mixed C₃/C₄ biome, particularly during the drier months. C₄ grasslands have been documented in comparable settings in the bordering area of northwestern Iran (Hatami and Khosravi, 2013). In these contexts, C₄ Chenopodiaceae species are known to persist until late autumn, and some may survive even into winter in protected microhabitats. Given this evidence it is highly unlikely for Maxta to be situated in a pure C_3_ environment year-round.

Furthermore, both Tornero at al 2016 and Janzen’s 2023 work on ancient and modern faunal assemblages indicate that animals grazing in lowland environments often display a C₄-enriched signal during the winter months, when C₄ plants persist in dry or sheltered microhabitats, and a more C₃-influenced signal during the summer only when herds access higher-elevation pastures where C₃ vegetation dominates. Further, Tornero et al 2016 also describe these lowlands as C3/C4 mixed environments in summer.

We have added the reference to Hatami and Khosravi (2013) in the revised manuscript to strengthen this argument.

**13.** Discussion. Line 653-654 you mention that part of the flock remained year-round at the site. However, lines 613-614 your interpretation of the isotopic data suggest the contrary.

We thank the reviewer for this observation. Our intention was to describe a dual herding system, in which zooarchaeological age-at-death profiles suggest that part of the flock may have remained year-round, while isotopic data indicate seasonal movement for another portion. These patterns are not mutually exclusive and together point to a flexible management strategy.

**14.** Figure 8 is quite problematic because it is a theoretical model that is not fully supported by your data. Its inclusion in the text gives the impression that it is the model for Kura-Araxes Maxta. Autumn birth and winter lactation are not proven. You have one individual out of five born in the summer. It could be an out-of-season born individual that survived. You don’t have enough data to suggest that there was an attempt for the herders to have two distinct birth seasons in order to have two lactation seasons. I don’t even speak about the goat as there are not enough reference data to precisely attribute Xo/X values to a birth season.

Thank you for your thoughtful comments regarding Figure 8, now Figure 7. We agree that the figure could be interpreted as overly definitive in its previous form. In response, we have made several revisions to clarify this.

- The figure title has been modified from *“Integrated model of seasonal pastoral strategies at Maxta I”* to *“Schematic representation of possible seasonal pastoral strategies at Maxta I”*
- The caption has been rewritten to emphasise the limitations of the seasonality of birth dataset: “*This schematic summarises the interplay between herd mobility, dual birthing seasons, architectural permanence, and resource use across the annual cycle. Drawing on stable isotope sequences, zooarchaeological data, and settlement features, the model highlights how caprines were seasonally moved between lowland and highland pastures, with distinct birthing and culling strategies tailored to fleece, meat, and dung production. Permanent domestic infrastructure at Maxta I anchored this flexible, seasonal economy. Autumn birth, winter lactation and archaeobotanical interpretations are presented as hypothetical scenarios that align with the available data but cannot be directly demonstrated due to the limited sample size (indicated with dashed lines and lighter colouring). The figure was created using* [*https://app.biorender.com/*](https://app.biorender.com/)*.*”
- Figure 7 has been visually adjusted so that autumn births and winter lactation and the archaeobotany are shown with dashed outlines and higher transparency to indicate the tentative nature of these inferences.
- Further we have addressed your concerns in the revised main text by clarifying the limitations of our dataset and outlining alternative scenarios (page 38, line 847): “*However, the limited dataset prevents assessment of whether the pattern represents deliberate staggering of births or occasional out-of-season events*.”

Mountain pasture in the summer is not supported by enough evidence (see my comment above about the 13C values expected from highland pastures).

Thank you for this comment. We have addressed this point earlier.

Sheep and goat flocks could have been used to manure the field. However, to evaluate the real impact of manuring, it would necessitate to have isotopic values from botanical remains from Maxta. To discuss the seasonality of sowing, again a detailed botanical study is needed.
The archaeobotanical evidence you mention line 763 are from the 2x2 sounding in 2006, how representative are they of the whole KA agriculture at Maxta?

Thank you for this comment. We have now integrated the published archaeobotanical results from Maxta I into the discussion of the seasonal agro-pastoral model. We have also removed fruits and legumes from Figure 7, as these categories are not supported by the available archaeobotanical evidence. We also highlight that the archaeobotanical study is small and spatially restrictive and should be seen as preliminary.

Page 40, lines 897:

*“Archaeobotanical evidence[66] from Maxta I is based on a small, spatially restricted sample and should be regarded as preliminary. The assemblage is dominated by barley (Hordeum vulgare), with minor bread/macaroni wheat (Triticum aestivum/durum), small quantities of wild and weedy taxa (e.g., Boraginaceae, Galium), and only limited evidence for the pulse bitter vetch (Vicia ervilia); no C₄ crops have been recovered. Barley was probably autumn-sown[257], a practice that could have benefited from winter dung deposition. Together with the isotopic results, this pattern is consistent with a possible synchronization of pastoral and agricultural cycles: caprines may have been moved to higher pastures after the spring growing season and early summer harvest, when lowland fields had dried to stubble and fresh forage was available in the highlands, and returned in autumn to fertilise fields. However, this scenario remains tentative and requires confirmation through broader archaeobotanical and plant isotopic evidence.”*

**15.** The limitations of the study are clearly described at the end of the article. However it is not enough to justify the discussion that is clearly aimed at justifying a theoretical model that doesn’t completely fit with the reality of the data.

We thank the reviewer for this observation. Our intention in the discussion was not to impose a fixed theoretical model, but to explore how the Maxta I results relate to existing interpretations of Kura–Araxes pastoral mobility and economy. We have clarified this point in the discussion and now state more explicitly that the proposed model is tentative and site-specific. (see page 42, lines: 956)

*“Our conclusions are drawn from a single site and should be seen as a contribution to an ongoing regional debate rather than a comprehensive explanation of Kura–Araxes lifeways. While the Maxta I evidence challenges some long-standing assumptions about mobility and settlement, it does not resolve the full variability of pastoral strategies across the Kura–Araxes world and will require testing against additional multi-proxy datasets from other sites.”*

**16.** The supplementary file “environmental background” also includes photographs of bone tools, ceramics, archaeological structures and zoomorphic figures. It is not clear to me why it is in the same document.

We thank the reviewer for this helpful suggestion. It has now been moved to a separate supplementary file titled S2 File.

**17.** Supplementary 2. Caucasus vegetation. It is not clear to me in which way the list of C4 and C3 plant species support the discussion of the paper. Concerning the δ13C values of C3 and C4 plants, the values reported from Rudov et al were obtained from plant from all over SW Asia. Is not necessarily helpful to decide whether Maxta animals consumed or not C4 plants. The values published by Shishlina and al are mostly coming from the Russian steppes, very far away from the Araxes basin and ecologically very different.

We thank the reviewer for this helpful comment. Supplementary Material 2 was included to provide context for interpreting the isotopic data by outlining how C₃ and C₄ plant taxa are distributed across the main vegetation zones of the Caucasus (semi-desert, steppe, montane, and sub-alpine). Because variations in δ¹³C values depend on the relative presence of C₃ and C₄ plants in different environments, this overview helps clarify the potential ecological settings in which the Maxta animals grazed.

The table brings together information not presented elsewhere in the main text, offering a concise reference for readers unfamiliar with the vegetation structure of the Caucasus. We believe it supports the discussion by illustrating the regional environmental background relevant to our isotopic interpretations.

As this material is descriptive rather than analytical, it was placed in the Supplementary Information to keep the main text focused while ensuring that the information remains accessible to readers interested in the broader ecological context.

We have already discussed the C₃ and C₄ isotopic cut-offs earlier. Concerning the use of *Rudov et al.* (2020), previous studies have typically relied on global average C₄ plant values, whereas we consider it more appropriate to use a regionally specific dataset from southwest Asia. This provides a closer environmental and ecological match to the vegetation patterns surrounding Maxta and therefore a more contextually grounded comparison.

Further we have added this issue to our limitation section on page 43, line 982:

*“A key limitation of this study is the absence of modern isotopic data for C₃ and C₄ plants from the immediate surroundings of Maxta. As a result, our comparative thresholds rely on published regional datasets. These may not capture the full local isotopic variability of plant communities. The values used here should therefore be regarded as approximate regional references rather than precise local baselines, and further sampling of modern vegetation in Nakhchivan is needed to refine these estimates.”*

**18.** Supplementary 5. Would be nice to add species and side.

We thank the reviewer for this helpful comment. We have updated now S6 Dataset, Table A, to include additional detail. The table now specifies species identifications (based on ZooMS), skeletal side, tooth element, and contextual information for each specimen.

**19.** Supplementary 9. I would be necessary to explain the abbreviations and the calculations

We thank the reviewer and have revised now S8 Dataset, to define all abbreviations (A, Ψ, M, D, d).
