# Supplementary material for: Seasonality and mobility: An Integrative framework for reconstructing Kura-Araxes pastoral systems at Maxta I, Nakhchivan
Source: PLoS One. 2026 Apr 16;21(4):e0346108. doi: 10.1371/journal.pone.0346108 (PMC13086362; doi:10.1371/journal.pone.0346108)
Supplement: Attachment — Submitted filename: Response_to_Reviewers_auresp_2.docx [file pone.0346108.s012.docx]

**PONE-D-25-44174R1**

**Seasonality and mobility: An Integrative framework for reconstructing Kura-Araxes pastoral systems at Maxta I, Nakhchivan**

We thank the Academic Editor and the reviewers for their detailed and constructive comments, which have greatly improved our manuscript. Below we provide a response to each point. Reviewer/Editor comments are shown in **black**, followed by our responses in **blue**.

**Reviewer #1:**

The second variant of the manuscript includes many changes, the outline of the paper presentation has been changed and some other changes have been incorporated. On the whole, the authors of the paper have provided explanation and clarification to most of the questions. The presented overall economic model used by one specific population group that lived at the Maxtra I settlement is based on development of pasture sheep/goat raising and cattle raising near the settlement; this population also cultivated crops. The revised text includes data of paleoarchaebotany. Generally speaking, this model is consistent with the economic model of the Caucasus inhabitants throughout the Middle Ages and the early 20th century where raising of ovicaprids played a key role.
The pending issue is to what extent raising of ovicaprids and use of adjacent pastures were correlated with the area of cultivated lands. It is clear that the author focused, mainly, on the primary role of extensive sheep/goat raising and related secondary productions; therefore, the geochemical data on the archaeozoological samples reflect a mobile nature of animal husbandry. However, the paper does not contain any multi-proxy analysis of the pasture/fodder system near the settlement and the system in the foothill area or their comparison; for this reason, the proposed interpretation remains a working hypothesis.
In the absence of the data on a significant role played by secondary products, first of all, data on the production of wool raw material, woolen cloths, and felt, I think that sheep and goats were raised to obtain meat, and, possibly, milk on a seasonal basis. I would like to note that ethnographic and modern data suggest that the lactation period of sheep in the Caucasus, probably, lasted for not more than two months or up to three months in some areas.
The issue of localization of winter pastures and fodder storage for winter has not yet been addressed. The authors analyze a potential environmental situation similar to the modern situation and think that the population could feed sheep/goats as well as cattle on pastures almost during the entire winter. The steppe landscape around Maxtra-1 seems to be consistent with this pasture strategy whereas foothill and mountainous pastures could be used during the warm period of the year. On the whole, while the authors mention that the steppe landscape and natural zone determined the type of the economic system developed by the Maxtra-I population, this statement should be spelled out with more clarity. Besides, the ethnography of the Caucasus highlanders suggests that shepherds often moved with their sheep from one area of mountainous summer pastures to another. Usually such movements lasted for 5-6 days because of overgrazing and time needed to restore pasture cover. This type of mobility should also be taken into account in developing the economic model; it is also important to take into account that, based on the ethnography of the Caucasus, only men of different ages were involved in such seasonal moves.
The proposed agro-pastoral model is based on new archaeozoological, ZooMS and isotope data, obtained for one site of the Kuro-Araxes culture and so far it remains to be the only model of this type. This model is the main outcome of the study and the basis for follow-up comparative analysis with other models that will be developed using materials from other sites. The paper is recommended for publication with minor changes and clarifications.

Thank you very much for this positive and constructive assessment. We have clarified in the revised manuscript (limitations section) that the proposed agro-pastoral model, especially the interpretation of mobility based on isotopic data, should be considered a working hypothesis in the absence of a full multi-proxy comparison of pasture and fodder systems. We also we retained a cautious interpretation of secondary products (wool/milk), given the current archaeological evidence.

**Minor comments**
**1.** Line 76 – more precise chronology of the Majkop culture of the northern Caucasus is 3650-3000 calBC. The dating of the human samples from the steppe region affected by a reservoir effect shifted the lower boundary backward to 3900 calBC, making the date older.

Thank you for alerting us to this. We have adjusted the date in text.

**2.** It is better to move lines 181-188 (description of Maxtra I location) up and put it before line 140 as well as descriptions of the ….. given further in the text.

Thank you for this comment. We have moved the description up.

**Reviewer #2:**

Thank you for the revised version of your manuscript. Although you answered to all my comments, some part of the discussion needs to be strengthened. I also have additional comments.

We are very grateful for your detailed assessment and the time you have taken to provide these helpful comments, which have significantly improved the manuscript. We have addressed each of your additional comments below.

**1.** L. 163 you should consider using the azeri spelling Çaqqallıqtəpə because there is absolutely nothing in books or internet about Chaggalligtepe

Thank you for this comment. We have updated the spelling throughout.

**2.** l. 453-454. There is a shift of approximately 5-6 month between the beginning of the crown formation and the enamel maturation. Hence the isotopic record rather reflects the life of the individual between its 6 and 18 months (see references 211 - 212 -226).

Thank you for this comment. We have added a sentence clarifying the ~5–6-month delay between crown formation onset and enamel maturation; therefore, the isotopic signal mainly reflects the individual’s life between ~6 and 18 months of age (~1 year).

*“There is a delay between enamel formation and isotopic signal incorporation [210,211]. Thus, each M2 provides an isotopic record of 6 – 18 months, approximately the first year of life”*

**3.** l. 488-499. The proposed threshold for pure C3 diet is rather low (although the upper range of the threshold in enamel is -8.58 for d13C). It is rightly stated that Janzen et al proposed a threshold at -6.7 (and not quoted Messana et al. a threshold at -7.4 in southern Europe see doi 10.1007/s12520-024-02116-z). The difference with your threshold is not discussed enough although it might highly impact the interpretation of the herd's diet.

Thank you for this comment. We acknowledge that published δ¹³C thresholds differ between studies (e.g., Janzen et al. and Messana et al.). However, thresholds derived from southern Europe are not necessarily transferable to our study area (We might be misunderstanding this part of your comment), and both Janzen et al.’s threshold and ours are inferred from the distribution of values rather than being based on local plant baseline data; therefore, neither can be considered more appropriate than the other. We are cautious about further comparing these cut-offs, as this could unintentionally imply that the Janzen threshold represents a broadly applicable reference for all Caucasus sites, which is not the case. Ultimately, the most robust approach would be to establish local baseline values for each study site/region (as e.g. done at Yeghegis-1). We have previously clarified this point in the manuscript, and we consider that the limitations are already clearly outlined in the Methods and study limitations sections. Our data will be published as open access and will be freely downloadable, so future researchers can readily use it for comparative studies, including reassessing interpretations as more robust, locally established cut-offs become available.

**4.** l. 552. If I understood well, 10% of the Bos primigenius bones are burnt. It means 1 bone (out of 10). On such frequency I would not comment the occurrence of burning. Same with the 9% of 11 large mammal remains. Considering that you studied approximately 25% of the assemblage, any significance of frequencies is difficult to assess.

Thank you for this helpful suggestion. We have removed the percentages from the text.

**5.** Fig. 4. You write Taxonomic abundance in % of the NISP but the graph shows n=410 (which includes unidentified remains). And the caprines are shown at approx 55% while in the table in supp 5 they make 68% of the NISP.

Thank you for altering us to this issue. The graph should say n=370, as it is based on the identifiable assemblage. We have therefore corrected this on Fig 4. They make up the following:

| Bos | 104 | 28.1 |
| --- | --- | --- |
| Sus | 2 | 0.5 |
| Caprines | 226 | 61.1 |
| Equus | 9 | 2.4 |
| Bos P. | 10 | 2.7 |
| CE | 2 | 0.5 |
| CC | 1 | 0.3 |
| Cervidae | 15 | 4.1 |
| Vulpes | 1 | 0.3 |
| Total | 370 | 100.0 |

**6.** L. 602-608. The text is not consistent with fig. 4 and Supplementary 6. The text and fig.4 mention 20 mandibles identified with Zoom (and 5 attributed to Ovis according to the morphology). In supp6, there are only 18 specimens. None is identified as sheep in the column F (morphology). And only 13 out of 18 are identified with Zoom (column G).

Thank you for alerting us to this. The discrepancy arises because not all specimens analysed for collagen were identified using ZooMS. ZooMS was applied primarily to the subset selected for sequential enamel sampling, as well as a small number of additional specimens (some of which were used for collagen analyses and some of which were not). We also acknowledge that there was an error in the total count reported: the correct total is 18 ZooMS-analysed specimens, but these are not identical to the specimens listed in Supplementary Table 6. We have thereby updated this in text and in Fig 4. Supplementary Table 6 includes only the specimens used for sequential enamel and/or collagen analyses and therefore does not include all specimens referred to in the main text. We have clarified this in the revised manuscript to guide the reader. The full spectra and complete ZooMS results table will be made accessible via Mendeley Data (doi: 10.17632/24dyjf7c5j.1)

*“We note that Table A in S6 Dataset lists only the specimens used for sequential enamel sampling and/or collagen analyses. ZooMS was performed on a wider set of mandibles, and therefore the total number of ZooMS-analysed specimens does not fully overlap with those listed in Table A. The full ZooMS results table and spectra are available on Mendeley Data doi: 10.17632/24dyjf7c5j.1.*”

**7.** L. 619. You write "table Sx". Indeed, the average values are missing from the supplementary data. It would be interesting for the reader to have these summary statistics. Boxplots to show the distribution of d13C and d18O in the different individuals would also be helpful.

Thank you for this helpful comment. We have added the summary statistics as Table E in S6 Dataset, together with a graph.

**8.** L. 679-680. In variation A.2, the lowest d13C value is -10.3. You mention highland pastures. It should be discussed why -10.3 could correspond to highland pastures.

Thank you for this comment. All patterns, including A.2, are discussed in detail in the manuscript. The value of -10.3‰ simply reflects a C3 signal occurring in the summer part of the sequence, which we consider unlikely for Maxta I, where we are confident that C4 plants were available during summer. Since C4 plants have been shown to occur in patches even in winter (see Janzen and Tornero, both cited in the manuscript), it would be highly unlikely for them to be absent during the warmest season.

**9.** L. 703-705. In pattern 3, you assume pasture in high altitude because d13C values around -8.5 / -9 look to low for you to correspond to the Araxes valley. However, you used the same argument to identify high-altitude summer pasture in pattern 1 with d13C values around -10. It is not the same high-altitude pastures then?
In general, in the section “Sequential enamel bioapatite δ18O and δ13C values”, the values used to identify high altitude pasture are not sufficiently discussed. You don’t even mention your own article about Yeghegis-1 where values for this site at 1,500 masl are around -11 for d13C. Also, I’m not convinced by your main argument for vertical mobility when you consider that any values below the C3/C4 threshold rules out local pastures around Maxta I. The Sharur plain is one of the greenest spot of Nakhchivan, it is among the main hay provider areas of Nakhchivan. It is watered by both the Arpaçay river and the ground water of the Araxes. One can imagine green fields around the site (2-3 km radius). It is not proven that grazing on C4 plant was inevitable around Maxta I.

Thank you for this comment. We do not agree with this point. High-altitude summer pasture would be expected to be C3-dominated, and the δ¹³C of plants is also expected to vary with both altitude and season. In contrast, Maxta I in summer represents a mixed C3/C4 environment, as demonstrated by multiple studies (cited in the manuscript). Therefore, the herders may have moved to pastures at different altitudes, or the variation could simply reflect seasonal changes in the local environment. You also mention the Sharur plain as one of the greenest areas, suggesting that green fields may have existed within a 2–3 km radius of the site, and thus that grazing on C4 plants was not necessarily inevitable around Maxta I. However, unless the Sharur plain is at an altitude too high for C4 plants, it is not clear why hay would not also include some C4 contribution, as these plants are seasonal and would be expected to grow during the warmest part of the year.

Concerning the environmental setting of the Sharur plain, we acknowledge that local conditions are heterogeneous. However, characterising it as a purely C3 environment would not be accurate. Present-day climatic data indicate an annual precipitation of approximately 300 mm, which classifies the area as semi-arid. Such conditions support a mixed C3/C4 biome, particularly during the drier months. C4 grasslands have been documented in comparable settings in the bordering area of northwestern Iran (Hatami and Khosravi, 2013). In these contexts, C4 Chenopodiaceae species are known to persist until late autumn, and some may survive even into winter in protected microhabitats. Given this evidence, it is highly unlikely for Maxta I to be situated in a purely C3 environment year-round.

Furthermore, both Tornero et al. (2016) and Janzen (2023), based on ancient and modern faunal assemblages, indicate that animals grazing in lowland environments may display a more C4-enriched signal during the winter months (when C4 plants persist in dry or sheltered microhabitats), and a more C3-influenced signal during the summer only when herds access higher-elevation pastures where C3 vegetation dominates. Tornero et al. (2016) also describe these lowlands as mixed C3/C4 environments in summer. Overall, an environment that supports C4 plants in winter is unlikely to lack them entirely during summer, given that these plants typically thrive under warmer conditions.

Yeghegis-1 has already been cited in the text, together with other relevant studies from the Caucasus. However, we note that directly comparing absolute δ¹³C and δ¹⁸O values across comparative datasets is not straightforward, given differences in chronology (spanning millennia), environmental context, and potential methodological variation (e.g., sampling strategy and preparation protocols). We therefore prefer to focus on the internal patterns within our dataset and interpret them within the local context of Maxta I.

**10.** L. 710 About the section “seasonality of birth”. I checked the raw data you provided in S6 and S7. I think that it would be necessary to provide the pictures of the sampled teeth, so that the reader can see how the sampling was made and can see the ERJ.
For example, I have a question about the distance to erj of the samples of MI 18. There should be a gap of at least 1 mm between two samples. Because the procedure is to measure the sample position on the upper ridge (occlusal direction). The drill is 1mm thick. How can you have 0.5 or 0.6mm between samples?
I think there is also a mistake in modelling MI 25. In S7, x0 for this tooth is 6.5 mm. but in the graph it is clear that the max dO values are rather between 15 and 20. Maybe the model proposed a negative amplitude. If you correct, you will have a very different x0/X and birth season for this individual.
Models for MI 16, MI 18 and MI 42 are tentative because you cannot be sure that the first point of the sequence is really a minimum. However, you might argue, than even with a longer period X, the X0/X value would not be so different in term of birth season.

Thank you for this comment. We have now added the images of the sampled teeth in S9 File. In our study, we measured sample positions along the lower ridge (*we have clarified this in the S9 File*), as either the upper or lower ridge can be used provided the approach is applied consistently. There is no paper that dictates one should measure from either the lower or upper ridge. In any case, a potential offset of ~1 mm in the distance-to-ERJ measurements would not significantly affect the estimated birth season. We have previously tested this by shifting all distances by 1 mm and re-running the model, which resulted in no meaningful change in the inferred birth season.

Thank you for alerting us to MI25. There indeed was a mistake in the x0 and the final season, which we have now corrected it in S7 and as well as on Figure 6.

We agree with your point that the models for MI16, MI18 and MI42 are tentative since the sampled sequences may not include a true minimum, and we have clarified this in the manuscript.

*“For MI16, MI18 and MI42, the birth-season estimates should be regarded as tentative because the sampled sequences may not capture a true δ¹⁸O minimum.”*

**11.** L. 726 Plotting the d15N and d13C in a graph would be easier for the reader. You could even use the graph you published in the Yeghegis-1 article to put these values into a broader context. Collagen values are not used enough in the discussion. Having both collagen and sequential enamel values from 10 individuals is an excellent idea. However, there is no discussion of the comparison of these two datasets. Are the pattern groups for enamel also visible in collagen? If not, what does it mean?

Thank you for this comment. We have now added the requested δ¹³C–δ¹⁵N plot as Figure 7. However, we have decided not to compare the bulk collagen dataset with the sequential enamel pattern groups, as these two proxies record different aspects of diet and mobility and operate at different temporal scales. Bulk collagen provides a time-averaged signal and does not capture seasonal variation, whereas sequential enamel reflects intra-annual changes during tooth formation. We have however tried to contextualise the collagen data with other regional studies in the text & graph.

**12.** L. 849. The focus on wool is not that well supported by Fig. 2 and even S2. You even wrote that pointed tools on ulna are common, ubiquitous, and probably not specific to textile production. In S2, if we compare with the tools published from Rabati, or even Godedzor (Palumbi et al 2021), I see very few strong markers of wool processing in Maxta I. The two objects at the bottom of fig. 2 C have some parallels with loom weights from Rabati. Bone spindle whorls made of a sawn and drilled bovine femur head seem to be a strong marker of KA textile production. Is there any from Maxta I? If yes you should indicate their frequency and provide pictures. They would be a much better argument for wool processing than the pointed tools made of ulna.

Thank you for this comment. We agree that some tool types, such as ulna tools, are not necessarily specific to textile production. However, we do not suggest that these tools were unrelated to textiles; rather, as also noted by Reviewer 1, they are likely multifunctional, and textile-related activities could reasonably be one of their possible uses, including the processing of both woollen fibres and plant fibres.

Regarding spindle whorls, Maxta I does in fact include bone whorls (femur). We have now included an image of this in Figure 3c. Although the exact frequency is not reported in the present dataset, we cite Safar’s book where they are discussed further.

In addition, S2 and Figure 3 include a needle, which is a more direct indicator of sewing/textile-related activity than pointed ulna tools. Figure 2 is intended to provide an overview of the material assemblage and site structure at Maxta I.

We have accordingly reduced our claims in the text to:
*“Further, Maxta I yielded numerous awls, needles, pins, borers and spindle whorls which may indicate some degree of involvement in textile-related production activities, though this interpretation remains tentative. [83] (S2 and Fig 3c).”*

**13.** L. 862. you should consider using the azeri spelling Çaqqallıqtəpə because there is absolutely nothing in books or internet about Chaggalligtepe

Thank you for this comment. We have updated the spelling throughout.

**14.** Bibliography :
There are some issues with references. I spotted a few but the rest should be checked.
References 51, 205, 206, 263 are incomplete.
46: not the right authors list.
47: incomplete author list

Thank you for alerting us to this. We have checked the bibliography and corrected these mistakes.
